# Faking in High-Stakes Personality Assessments: A Response-Time-Based Latent Response Mixture Modeling Approach

**DOI:** 10.1177/00131644261422169

**Published:** 2026-03-18

**Authors:** Timo Seitz, Esther Ulitzsch

**Affiliations:** 1 University of Mannheim, Germany; 2 Centre for Educational Measurement (CEMO), University of Oslo, Norway

**Keywords:** faking, mixture model, response times, item response theory

## Abstract

When personality assessments are employed in high-stakes contexts, there is the risk that test-takers provide overly positive descriptions of themselves. This response bias is known as faking and has often been addressed in latent variable models through an additional dimension capturing each test-taker’s faking degree. Such models typically assume a homogeneous response strategy for all test-takers, with substantive traits and faking jointly influencing responses to all items. In this article, we present a latent response mixture item response theory (IRT) model of faking that accounts for changes in test-takers’ response strategies over the course of the assessment. The model translates theoretical considerations about test-taker behavior into different model components for item responses and corresponding item-level response times (RT), thereby allowing to account for, identify, and investigate different faking-related response strategies on the person-by-item level. In a parameter recovery study, we found that the model parameters can be estimated well under realistic conditions. Also, we applied the model to an empirical dataset (*N* = 1,824) from a job application context, showcasing its utility in real high-stakes assessment data. We conclude the article by discussing the role of the model for psychological measurement as well as substantive research.

## Introduction

Personality questionnaires based on self-report are commonly used in high-stakes contexts like personnel selection or college admission, as personality traits have repeatedly been shown to predict relevant outcomes such as job performance or academic success (e.g., Poropat, 2009; Sackett & Walmsley, 2014). Nevertheless, employing self-report personality tests in contexts where the assessment results have important consequences for test-takers holds the risk that test-takers portray themselves in an overly positive light. This behavior is commonly referred to as *faking*, and its effects on the psychometric properties of a test are well documented (Ziegler et al., 2011). For instance, faking leads to negatively (positively) skewed distributions of items and scales measuring desirable (undesirable) attributes (e.g., Hu & Connelly, 2021). Also, it alters rank orders of test-takers (e.g., Mueller-Hanson et al., 2003), which impacts decisions where test-takers are selected based on their test scores. Most importantly, however, faking distorts construct validity of personality measures as it introduces an additional source of systematic variance. This leads to inflated inter-item and inter-scale correlations, diminishing the test’s factor structure (e.g., Schmit & Ryan, 1993) and rendering the obtained scores inappropriate for their intended purpose of usage (Messick, 1989). Hence, it is vital to have psychometric tools that effectively account for faking, especially in the context of high-stakes assessments. Such methods can be used to detect and statistically control for faking, but also to study the associated response process. Whereas the former can pay dividends when data have already been collected, the latter is important for advancing the theoretical understanding of faking, which can in turn be used to develop assessment tools that are less susceptible to faking.

Faking is especially problematic because of its heterogeneity. This is evidenced by studies investigating the prevalence of faking in job applications. Griffith and Converse (2011) reviewed the literature on this topic and concluded that roughly 30% (±10%) of applicants engage in faking behavior. That is, some test-takers indeed respond in a way that facilitates their appearance, whereas others do not. This is supported by Robie et al. (2007), who conducted a think-aloud study of test-takers responding to a personality test in a high-stakes condition. Using a verbal protocol analysis, they found one group of test-takers being fully honest in their responses, one group considering both their actual personality and the criteria of an “ideal” applicant, as well as one group exclusively responding based on “ideal” applicant considerations. In a related study, Röhner et al. (2025) identified as many as 35 different behaviors constituting distinct faking strategies.

To account for heterogeneity in faking, several model-based approaches have been proposed. Most of them either quantify the degree of faking on a latent continuum (e.g., Hendy et al., 2021; Klehe et al., 2012; Seitz et al., 2024; Seitz, Spengler, & Meiser, 2025) or assign test-takers to latent classes that represent qualitatively different response behaviors related to faking (e.g., Zickar et al., 2004). Other models combine quantitative and qualitative conceptualizations of faking to better describe the nature of the construct (Seitz, Alagöz, & Meiser, 2025; Ziegler et al., 2015). What most models have in common, however, is that they treat faking as a person variable that is constant across test items (see Böckenholt, 2014; Brown & Böckenholt, 2022; for exceptions). Yet, one can for multiple reasons question whether it is appropriate to conceptualize faking as constant throughout the entire test: First, faking is a complex interaction of person and situation characteristics, such as ability, opportunity, and motivation to fake (Tett & Simonet, 2011). Motivation to fake in personnel selection, for instance, may vary between items when some items are perceived as more instrumental than others to elevate the chance of being hired in a given job context (e.g., Ellingson & McFarland, 2011). Second, lying research has shown that people tend to behave dishonestly to the extent that they profit, but not to the extent that they damage their self-concept of being an honorable person (Mazar et al., 2008). If one has nothing to conceal at a particular item such that misreporting would not pay off, it can hence be expected that people will not engage in faking (Tourangeau & Yan, 2007). Third, test-takers have conflicting goals in high-stakes assessments. On the one hand, they want to impress a prospective employer. On the other hand, conveying a credible impression and staying true to oneself have also been identified as important motives of test-taking behavior (Kuncel et al., 2011). All these arguments suggest it is more plausible to assume that faking also varies between items than to treat it as a constant person variable.

In this work, we present a mixture item response theory (IRT) model that allows to account for, identify, and investigate different faking-related response strategies on the person-by-item level. The model we propose translates theoretical considerations about test-taker behavior into different model components (i.e., latent classes) for item responses and corresponding item-level response times (RT). Modeling RTs serves two purposes: First, from a statistical perspective, incorporating additional behavioral data into the model facilitates class separation. Second, from a substantive perspective, doing so allows investigating the response process behind faking in a sophisticated manner. It, for instance, allows examining the question of whether faking actually increases or decreases RTs. Also, the model as a whole can be used to identify what items are especially susceptible to faking, or to study relationships of substantive person characteristics with different faking tendencies. The inclusion of RTs thus constitutes an important extension over existing models allowing for varying faking behavior across items. Furthermore, our proposed model has the advantage of being able to account for nonmonotonic faking effects (see Figure 2) as well as including an additional faking class (a more detailed description of differences to related models can be found below).

## Proposed Model

**Figure 1.**
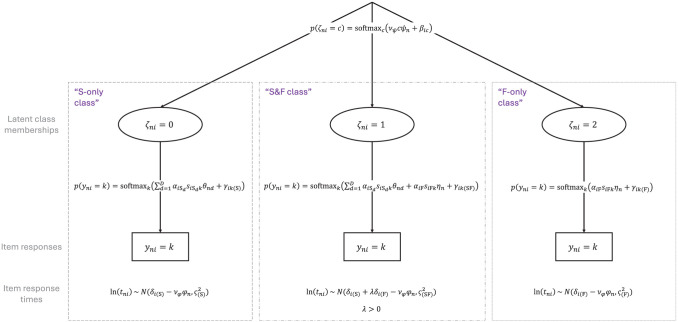
The Proposed Response-Time-Based Latent Response Mixture Model of Faking.

The model we present builds on work by Seitz, Alagöz, and Meiser (2025), who modeled faking using three latent classes related to distinct response strategies test-takers can employ in high-stakes assessments. We extend their model by adding an item-wise mixture component and combine it with approaches that make use of RT data to account for disengaged and careless responding in the context of cognitive and non-cognitive assessments (e.g., Ulitzsch et al., 2020; Ulitzsch, Pohl, et al., 2022). The full model is displayed in Figure 1.

### Model Components

#### Item Response Models

Similar to Seitz, Alagöz, and Meiser (2025), we assume that item responses are mixtures of three response strategies (see also the study by Robie et al., 2007, described above). The first response strategy (“S-only class”) is a strategy where test-takers respond according to their substantive traits, that is, respond honestly and do not align responses with social desirability. The second response strategy (“S&F class”) is a strategy where item responses are a function of test-takers’ substantive traits as well as a faking dimension. Responses thus represent edited versions of honest responses depending on the faking degree of a test-taker. The third response strategy (“F-only class”) is a strategy where item responses are independent of test-takers’ substantive traits. Instead, responses are solely influenced by the faking dimension.

Item responses $y_{\mathrm{ni}}\in \{0,\;1,\;\ldots ,\;k,\;\ldots ,\;K\}$ are modeled using different IRT models. Technically, the different models are constrained versions of Falk and Cai’s (2016) parameterization of the multidimensional nominal response model (MNRM; Takane & de Leeuw, 1987) depending on response strategy use. Strategy use per item is represented as a latent class $\zeta_{\mathrm{ni}}\in \{0,\;1,\;2\}$ . In the “S&F class” ( $\zeta_{\mathrm{ni}}=1$ ), where responses are a function of substantive traits and faking, the probability of person n choosing response category k on item i is modeled as

(1) $$p(y_{\mathrm{ni}}=k\;|\;\zeta_{\mathrm{ni}}=1)=\mathrm{softma}x_{k}\left(\sum_{d=1}^{D}\alpha_{iS_{d}}s_{iS_{d}k}\theta_{\mathrm{nd}}\;+\;\alpha_{iF}s_{iFk}\eta_{n}\;+\;\gamma_{\mathrm{ik}\left(\mathrm{SF}\right)}\right).$$

**Figure 2.**
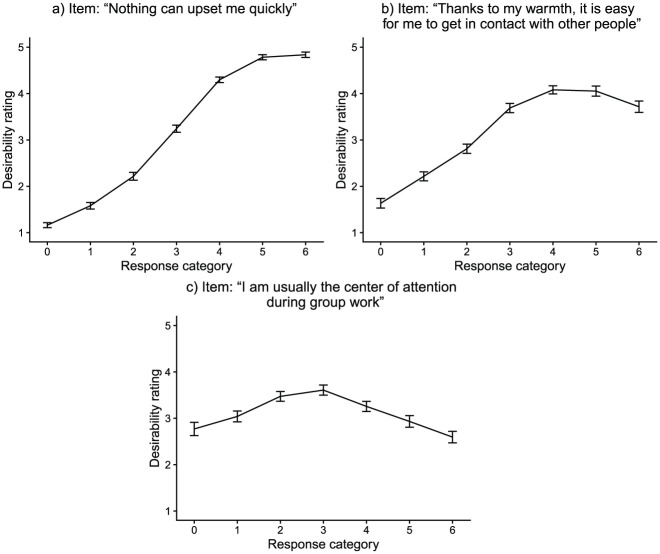
Desirability Trajectories of Three Exemplary Items From the Empirical Demonstration. *Note.* Mean desirability ratings are based on *N* = 74 participants (data from Seitz, Alagöz, & Meiser, 2025, Pilot Study 2). Error bars represent the standard error of the mean.

This softmax function (aka multinomial logistic function) converts a vector of K+1 real-valued category propensities into a probability distribution. Category propensities depend on a vector of person n ’s scores on D measured substantive trait dimensions ( $\theta_{n}$ ), person n ’s faking score $\eta_{n}$ , a (K+1) -dimensional vector of class-specific item-category intercepts $\gamma_{i\left(\mathrm{SF}\right)}$ , a D -dimensional vector of item slopes of substantive traits $\alpha_{iS}$ , an item slope of faking $\alpha_{iF}$ , and a ((D+1)×(K+1)) -dimensional matrix of scoring weights $S_{i}$ . Substantive trait scores $\theta_{\mathrm{nd}}$ represent the level of person n on the trait of interest d , faking scores $\eta_{n}$ indicate the degree to which person n aligns his or her responses with the items’ desirability characteristics.^1^ Item slopes $\alpha_{\mathrm{id}}$ represent the relation between item i and dimension d , whereas scoring weights $s_{\mathrm{idk}}$ reflect the item-specific relation between category k and dimension d . Because of this property of scoring weights, scoring weights are used to specify the to-be-modeled latent dimensions based on theoretical assumptions. For a substantive trait that is measured by a particular item, a scoring weight vector of evenly spaced integers is specified, as higher response categories should go along with higher substantive trait levels. In contrast, for substantive traits not measured by an item, scoring weights are set to 0. Analogously, scoring weights are specified for a faking dimension. Since scoring weights code relationships between categories and dimensions, scoring weights of faking can be specified such that they reflect the desirability characteristics of individual items (Seitz et al., 2024; Seitz, Spengler, & Meiser, 2025). To set scoring weights of faking in empirical contexts, one possibility is to use desirability ratings of item-category combinations from a pilot study (see Figure 2). Note that, because scoring weights are item- and category-specific, this modeling approach of faking allows accounting for various kinds of item desirability characteristics within a test. Thus, not only monotonic desirability trajectories (where desirability increases/decreases monotonically with higher categories; see Figure 2a) but also nonmonotonic ones (where the category of highest desirability is not an extreme category; see Figures 2b and c) can be modeled (cf. Kuncel & Tellegen, 2009).

In both the “S-only class” and “F-only class,” theory-motivated constraints are imposed on item slope parameters (Alagöz & Meiser, 2024; Seitz, Alagöz, & Meiser, 2025). Because responses are not influenced by faking in the “S-only class” ( $\zeta_{\mathrm{ni}}=0$ ), slopes of faking are set to 0 in this class, such that category propensities do not depend on faking scores:

(2) $$p(y_{\mathrm{ni}}=k\;|\;\zeta_{\mathrm{ni}}=0)=\mathrm{softma}x_{k}\left(\sum_{d=1}^{D}\alpha_{iS_{d}}s_{iS_{d}k}\theta_{\mathrm{nd}}\;+\;\gamma_{\mathrm{ik}\left(S\right)}\right).$$

This model is equivalent to a multidimensional generalized partial credit model (MGPCM; Muraki, 1992). By contrast, in the “F-only class” ( $\zeta_{\mathrm{ni}}=2$ ), slopes of substantive traits are set to 0. Category propensities are thus independent of substantive trait scores, implying a unidimensional nominal response model (NRM; Bock, 1972) with specified scoring weights of faking:

(3) $$p(y_{\mathrm{ni}}=k\;|\;\zeta_{\mathrm{ni}}=2)=\mathrm{softma}x_{k}\left(\alpha_{iF}s_{iFk}\eta_{n}\;+\;\gamma_{\mathrm{ik}(F)}\right).$$

Note that non-fixed slopes in this model are class-invariant, whereas item-category intercepts are class-specific. This has the purpose of measuring the same latent variables across classes, while at the same time being able to capture different item response distributions in the three classes. For model identification, the intercept of the first category is fixed to 0 for all items in all classes.

#### Item Response Time Models

Research has repeatedly demonstrated RT differences associated with response sets like honest responding, responding under conditions of heightened desirability concerns, or instructed faking (e.g., Holden et al., 1992; Holtgraves, 2004). To utilize this information in our mixture modeling approach, we specify different RT models associated with the different response strategies (see Ulitzsch et al., 2020; Ulitzsch, Pohl, et al., 2022, for similar approaches). Specifically, RTs $t_{\mathrm{ni}}$ are modeled through log-normal models featuring a person speed parameter $\varphi_{n}$ and item time intensity parameters $\delta_{i}$ (van der Linden, 2006). Speed is part of a multivariate normal distribution together with the other person parameters of the model (van der Linden, 2007). Time intensities reflect the predicted log-RTs for test-takers with a speed score of 0 (which represents the latent mean in our case).

To capture mean differences in RTs between classes, item time intensities are class-specific. Regarding the question of whether honest responding or faking takes longer, the literature is inconsistent. Some studies found faking to be associated with reduced RTs (e.g., Holden, 1995; Holden et al., 1992; Hsu et al., 1989), while other studies found faking to increase RTs (e.g., Fine & Pirak, 2016; Holtgraves, 2004; Walczyk et al., 2003). Hence, no constraints are put on time intensities in the “S-only class” ( $\zeta_{\mathrm{ni}}=0$ ) and “F-only class” ( $\zeta_{\mathrm{ni}}=2$ ):

(4) $$\ln\left(t_{\mathrm{ni}}\;|\;\zeta_{\mathrm{ni}}=0\right)\sim \;N(\delta_{i\left(S\right)}-\nu_{\varphi }\varphi_{n},\;\varsigma_{\left(S\right)}^{2}),$$

(5) $$\ln\left(t_{\mathrm{ni}}\;|\;\zeta_{\mathrm{ni}}=2\right)\sim \;N(\delta_{i\left(F\right)}-\nu_{\varphi }\varphi_{n},\;\varsigma_{\left(F\right)}^{2}).$$

The parameter $\nu_{\varphi }$ is an item-invariant speed slope, and $\varsigma_{\left(S\right)}^{2}$ and $\varsigma_{\left(F\right)}^{2}$ denote class-specific residual variances of log-RTs. In the “S&F class,” time intensities are constrained to be a function of “S-only class” time intensities $\delta_{i(S)}$ and “F-only class” time intensities $\delta_{i(F)}$ , as responses in this class are based on substantive traits *and* entail response editing according to the items’ desirability characteristics:

(6) $$\ln\left(t_{\mathrm{ni}}\;|\;\zeta_{\mathrm{ni}}=1\right)\sim \;N(\delta_{i\left(S\right)}\;+\;\lambda \delta_{i\left(F\right)}-\nu_{\varphi }\varphi_{n},\;\varsigma_{\left(\mathrm{SF}\right)}^{2}),$$

with $\varsigma_{\left(\mathrm{SF}\right)}^{2}$ denoting the class-specific residual variance of log-RTs, and λ being a proportionality constant on $\delta_{i(F)}$ . Because both response processes are involved in the “S&F class,” we expect mean RTs in this class to be longer than mean RTs in both the “S-only class” and “F-only class” (Walczyk et al., 2003). To encode this assumption, λ is constrained to be positive. It hence reflects the extent to which faking along with substantive trait responding increases RTs compared to pure substantive trait responding.^2^

Note that, in the proposed model, RTs are modeled as indicators of class membership as opposed to predictors of class membership (see Nagy & Ulitzsch, 2022; cf. Ulitzsch et al., 2020; Ulitzsch, Pohl, et al., 2022). That is, RTs are reflections of strategy use and contribute to the classification of responses by their relative typicality for the respective class. Since RT distributions are allowed to overlap, classification is not based on fixed RT cutoffs. While “S&F class” responses are assumed to take on average longer than their single-process counterparts, the overlap of RT distributions allows also short responses to be classified as “S&F class” responses, as well as also long responses to be classified as “S-only class” or “F-only class” responses.

#### Latent Response Model

Test-takers’ latent class memberships $\zeta_{\mathrm{ni}}$ are not observable. However, class memberships determine the measurement model of individual responses and RTs, and thus represent latent response variables (see Maris, 1995; cf. Ulitzsch et al., 2020; Ulitzsch, Pohl, et al., 2022; Ulitzsch, Yildirim-Erbasli, et al., 2022). To model item-wise class membership, we treat the three response strategies as ordinal, with the “F-only class” reflecting the most extreme self-presentation strategy, the “S&F class” reflecting an intermediate self-presentation strategy, and the “S-only class” reflecting the least pronounced self-presentation strategy. Another person parameter, strategy inclination $\psi_{n}$ , as well as item-class intercepts $\beta_{\mathrm{ic}}$ determine the propensity of being a member of class c on item i . A softmax function transforms the propensities into probabilities, which are the mixing proportions in the proposed person-by-item mixture model:

(7) $$p(\zeta_{\mathrm{ni}}=c)=\mathrm{softma}x_{c}\left(\nu_{\psi }c\psi_{n}\;+\;\beta_{\mathrm{ic}}\right).$$

This model represents a partial credit model (PCM; Masters, 1982) with an item-invariant strategy inclination slope $\nu_{\psi }$ . Conceptually, strategy inclination can be interpreted as the tendency of a person to use a more pronounced self-presentation strategy, that is, prefer the “F-only class” over the “S&F class” and the “S&F class” over the “S-only class.” Item-class intercepts can be interpreted as item easiness parameters associated with a particular strategy. For identification, the intercept of the first class (“S-only class”) is fixed to 0 for all items.

The D+3 person parameters of the proposed model are assumed to follow a joint multivariate normal distribution with expectation vector μ and variance-covariance matrix Σ :

(8) $$\mu =\left(\begin{array}{c} \mu_{\theta_{1}}\\ \vdots \\ \mu_{\theta_{d}}\\ \vdots \\ \mu_{\theta_{D}}\\ \mu_{\eta }\\ \mu_{\varphi }\\ \mu_{\psi } \end{array}\right)\;\mathrm{and}\;\Sigma =\left(\begin{array}{c} \sigma_{\theta_{1}}^{2}\;\;\;\;\;\;\;\;\;\;\;\;\;\;\\ \vdots \;\;\ddots \;\;\;\;\;\;\;\;\;\;\;\;\\ \sigma_{\theta_{1}\theta_{d}}\;\;\vdots \;\;\sigma_{\theta_{d}}^{2}\;\;\;\;\;\;\;\;\;\;\\ \vdots \;\;\vdots \;\;\vdots \;\;\ddots \;\;\;\;\;\;\;\;\\ \sigma_{\theta_{1}\theta_{D}}\;\;\vdots \;\;\sigma_{\theta_{d}\theta_{D}}\;\;\vdots \;\;\sigma_{\theta_{D}}^{2}\;\;\;\;\;\;\\ \sigma_{\theta_{1}\eta }\;\;\cdots \;\;\sigma_{\theta_{d}\eta }\;\;\cdots \;\;\sigma_{\theta_{D}\eta }\;\;\sigma_{\eta }^{2}\;\;\;\;\\ \sigma_{\theta_{1}\varphi }\;\;\cdots \;\;\sigma_{\theta_{d}\varphi }\;\;\cdots \;\;\sigma_{\theta_{D}\varphi }\;\;\sigma_{\eta \varphi }\;\;\sigma_{\varphi }^{2}\;\;\\ \sigma_{\theta_{1}\psi }\;\;\cdots \;\;\sigma_{\theta_{d}\psi }\;\;\cdots \;\;\sigma_{\theta_{D}\psi }\;\;\sigma_{\eta \psi }\;\;\sigma_{\varphi \psi }\;\;\sigma_{\psi }^{2} \end{array}\right)\;.$$

To identify the scale of latent variables, latent means are set to 0 and latent variances to 1, such that latent covariances represent latent correlations. Assuming conditional independence of item responses and RTs, the model’s joint likelihood of the data, marginalized over the three latent classes, can be denoted as

(9) $$L=\overset{N}{\underset{n=1}{\Pi }}\overset{I}{\underset{i=1}{\Pi }}\begin{array}{c} (p\left(\zeta_{\mathrm{ni}}=0\;|\;\psi_{n},\;\nu_{\psi },\;\beta_{\mathrm{ic}}\right)\;p\left(y_{\mathrm{ni}}\;|\;\theta_{n},\;\alpha_{iS},\;S_{i},\;\gamma_{i\left(S\right)}\right)\;f\left(t_{\mathrm{ni}}\;|\;\varphi_{n},\;\nu_{\varphi },\;\delta_{i\left(S\right)},\;\varsigma_{\left(S\right)}^{2}\right)\;+\;\\ p\left(\zeta_{\mathrm{ni}}=1\;|\;\psi_{n},\;\nu_{\psi },\;\beta_{\mathrm{ic}}\right)\;p\left(y_{\mathrm{ni}}\;|\;\theta_{n},\;\eta_{n},\;\alpha_{iS},\;\alpha_{iF},\;S_{i},\;\gamma_{i\left(\mathrm{SF}\right)}\right)\;f\left(t_{\mathrm{ni}}\;|\;\varphi_{n},\;\nu_{\varphi },\;\delta_{i\left(S\right)},\;\delta_{i(F)},\;\lambda ,\;\varsigma_{\left(\mathrm{SF}\right)}^{2}\right)\;+\;\\ p\left(\zeta_{\mathrm{ni}}=2\;|\;\psi_{n},\;\nu_{\psi },\;\beta_{\mathrm{ic}}\right)\;p\left(y_{\mathrm{ni}}\;|\;\eta_{n},\;\alpha_{iF},\;S_{i},\;\gamma_{i\left(F\right)}\right)\;f\left(t_{\mathrm{ni}}\;|\;\varphi_{n},\;\nu_{\varphi },\;\delta_{i\left(F\right)},\;\varsigma_{\left(F\right)}^{2}\right)) \end{array}$$

where N is the total number of persons, I is the total number of items, and f(...) is the log-normal density of RTs.

### Model Estimation

The proposed person-by-item mixture model can be estimated with a Bayesian Markov chain Monte Carlo (MCMC) procedure. We used the software *JAGS* (version 4.3.2; Plummer, 2017) for model estimation, accessed through the *R* environment (version 4.4.3) using the package *runjags* (Denwood, 2016). To process MCMC outputs, we employed the packages *coda* (Plummer et al., 2006) and *MCMCvis* (Youngflesh, 2018). The JAGS syntax as well as R code for estimating the model can be found at https://osf.io/crmv4/.

We used the following prior distributions for the different model parameters: Slope parameters were drawn from positively truncated normal priors ( $\alpha_{iS_{d}},\;\alpha_{iF},\;\nu_{\varphi },\;\nu_{\psi }\;\sim \;N^{+}\left(0,\;2^{2}\right)$ ). Class-specific item-category intercepts in the item response model components were sampled from uncensored normal priors ( $\gamma_{\mathrm{ik}\left(S\right)},\;\gamma_{\mathrm{ik}\left(\mathrm{SF}\right)},\;\gamma_{\mathrm{ik}\left(F\right)}\;\sim \;N\left(0,\;4^{2}\right)$ ), with the intercept of the first category fixed to 0 for model identification. The same prior was used for item-class intercepts in the latent response model component ( $\beta_{\mathrm{ic}}\;\sim \;N(0,\;4^{2})$ , intercept of the first class fixed to 0 for identification). For “S-only class” and “F-only class” item time intensities in the item RT model components, normal priors were used as well, with the empirical mean of log-RTs across persons and items serving as prior center ( $\delta_{i\left(S\right)},\;\delta_{i\left(F\right)}\;\sim \;N(\bar{\ln(t_{\mathrm{ni}})},\;1^{2})$ ). Since the “S&F class” proportionality constant was constrained to be greater than 0 but was not expected to take on large values, a positively truncated normal prior with a rather small variance was employed for this parameter ( $\lambda \;\sim \;N^{+}(0,\;0.5^{2}$ )). For residual standard deviations of log-RTs, standard half-Cauchy priors were used, which are positively truncated central *t*-distributions with 1 degree of freedom ( $\varsigma_{\left(S\right)},\;\varsigma_{\left(\mathrm{SF}\right)},\;\varsigma_{\left(F\right)}\;\sim \;t^{+}(1)$ ). The prior for person parameters was a multivariate normal distribution ( $\left(\theta_{n},\;\eta_{n},\;\varphi_{n},\;\psi_{n})\;\sim \;\mathrm{MVN}(\mu ,\;\Sigma \right)$ ), with μ=0 and Σ as a variance-covariance matrix with unit variances for identifying the scale of latent variables. Covariances, which reflected correlations, had uniform priors of *U*(−1, 1). To sample class membership $\zeta_{\mathrm{ni}}$ , values were drawn from a categorical distribution $\mathrm{Cat}(\pi_{\mathrm{ni}})$ , with $\pi_{\mathrm{ni}}$ being a vector of person- and item-specific class probabilities resulting from Equation 7 at the respective MCMC iteration.

We considered means of posterior distributions as point estimates of model parameters. In addition, we derived posterior class probabilities and class proportions estimates. Since class probabilities are a direct function of the parameters from the latent response model, posterior class probabilities can be computed by plugging the samples of the latent response model parameters into Equation 7 for each non-discarded MCMC iteration before aggregating across iterations. To get class proportion estimates, mean class probabilities across persons and iterations can be calculated, either per item or aggregated across items. It is worth noting that class labels in the proposed model are not arbitrary since the measurement models of the three classes are explicitly specified. This confirmatory specification of latent classes avoids the issue of label switching, which is often encountered when estimating mixture models in a Bayesian framework (Jasra et al., 2005).

### Differences to Related Faking Mixture Models

As elaborated above, most faking models do not account for a faking process that varies between items. However, there are some models that do allow for inter-item variation in how item responses are (not) affected by substantive traits and faking. Böckenholt (2014), for instance, presented a model of motivated misreports that is characterized by possible response editing before providing answers to sensitive survey questions (see also Leng et al., 2020). This model includes for every item a dichotomous latent class variable indicating if a test-taker edits his or her retrieved response. In the case of editing, a transition function models the selection of response categories that are more desirable than the category that would have been chosen without editing. Though theoretically appealing, the model is limited to only one substantive trait dimension. Also, response editing is restricted to go in the direction of high (low) categories if the measured substantive trait is generally desirable (undesirable).

Another model allowing for switches between response strategies over the course of the test is Brown and Böckenholt’s (2022) model of intermittent faking. This model assumes that item responses are mixtures of honest and faked responses, with honest (“real”) responses influenced by substantive traits and faked (“ideal”) responses influenced by a faking factor. Whether responses are honest or faked is modeled through an editing factor and item characteristics. This model is not limited to a single substantive trait; however, its frequentist estimation curbs the number of dimensions that can practically be modeled. Also, the model adopts a structural equation modeling (SEM) approach, which was empirically demonstrated using total scale scores as indicator variables and not individual items. In addition, the possibility of nonlinear and nonmonotonic item desirability trajectories (Kuncel & Tellegen, 2009; Seitz et al., 2024; see Figure 2) is not directly taken into account. Furthermore, the model does not include a class where substantive traits *and* faking influence item responses. However, such a class is conceivable because studies have found faking to operate at the editing stage of item responding and not at the retrieval stage (Holtgraves, 2004; Sudman et al., 1996; Walczyk et al., 2003). Robie et al. (2007) also showed that a considerable share of test-takers in high-stakes assessments refer to both their true personality and the ideal applicant when giving item responses.

Along with addressing several limitations of related mixture models of faking (modeling multiple substantive traits, nonmonotonic faking effects, and an “S&F class”), the proposed model also incorporates item-level RTs. As elaborated above, this can provide valuable insights into the cognitive processes associated with faking. The use of collateral data can also be expected to facilitate the estimation of such a person-by-item mixture model, where the information for class assignment is very sparse compared to a mixture model with constant class membership per person.

## Parameter Recovery Study

To evaluate the proposed model under realistic conditions, we conducted a parameter recovery study. The purpose of this study was twofold: a) to examine the model’s ability to accurately recover parameters and b) to compare it to the performance of less complex models.

### Data Generation and Fitted Models

For emulating realistic conditions, data-generating values for item responses and item RTs were in line with parameter estimates from the empirical demonstration below. Resembling our empirical dataset, data from a test measuring 3 substantive traits with 10 items each on a 7-point Likert scale were simulated using the R packages *MASS* (Venables & Ripley, 2002) and *extraDistr* (Wolodzko, 2023). For the parameter recovery study, a sample size of *N* = 500 was considered. 50 independent replications were performed. Further details on the data generation procedure can be found in the Online Supplement.

Each simulated dataset was analyzed using the proposed person-by-item mixture model including RTs. In addition, four less complex models were fitted to every dataset: a person-by-item mixture model not accounting for RTs, a person mixture model accounting for RTs, a person mixture model not accounting for RTs, as well as a non-mixture model. Comparing models with and without RTs allowed investigating how the inclusion of RTs improves model estimation in terms of parameter recovery and class separation. Comparing person-by-item and person mixture models allowed investigating the consequences of disregarding varying strategy use across items. Comparing mixture and non-mixture models allowed examining the effect of assuming a single response strategy across persons and items.

The non-mixture model was an MNRM accounting for substantive traits and faking with specified scoring weights (see Equation 1). All mixture models included the three described latent classes; however, the two person mixture models did not have the latent response model component. Instead, latent class membership in the person mixture models was only a person variable sampled from a categorical distribution Cat(π) with a flat Dirichlet hyperprior ( π~Dir(1) ). The mixture models without RTs were equivalent to their RT counterparts, with the exception of not having the item RT model components. All model syntaxes are available at https://osf.io/crmv4/. Scoring weights were specified as in the data generation. For the Bayesian estimation of each model, 4 parallel MCMC chains were run with 4,000 burnin and 10,000 regular iterations.^3^ Model convergence was assessed based on $\hat{R}$ (Gelman & Rubin, 1992), with a model considered as converged if all model parameters had $\hat{R}$ values below 1.1.

### Results of the Parameter Recovery Study

The person-by-item mixture model with RTs converged in 49 out of 50 replications (98%). The person-by-item mixture model without RTs, however, only converged in 37 replications (74%). With regard to the two person mixture models, the model with RTs converged in 39 replications (78%) and the model without RTs in 48 replications (96%). The non-mixture model converged in all 50 replications (100%).

**Figure 3.**
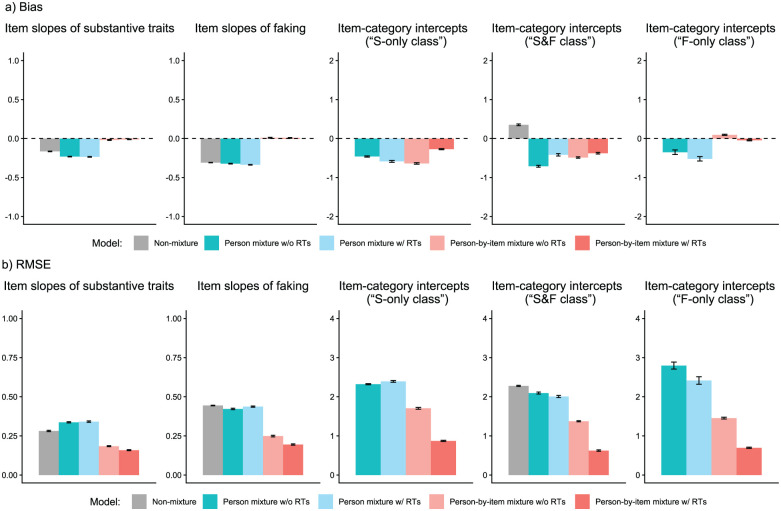
Parameter Recovery Study: Recovery of Item Response Model Parameters. *Note.* Values reflect the mean bias (Panel a) or root mean square error (RMSE; Panel b) of estimated parameters across replications. For mixture models, the respective class-specific intercept estimates are considered. Error bars represent the standard error of the mean. RT = response time.

**Figure 4.**
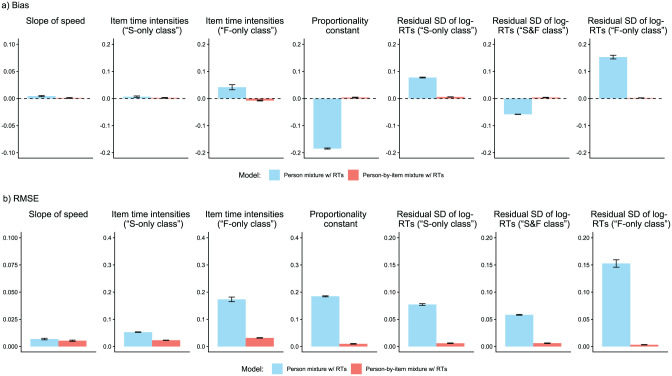
Parameter Recovery Study: Recovery of Item Response Time Model Parameters. *Note.* Values reflect the mean bias (Panel a) or root mean square error (RMSE; Panel b) of estimated parameters across replications. Error bars represent the standard error of the mean. SD = standard deviation; RT = response time.

**Figure 5.**
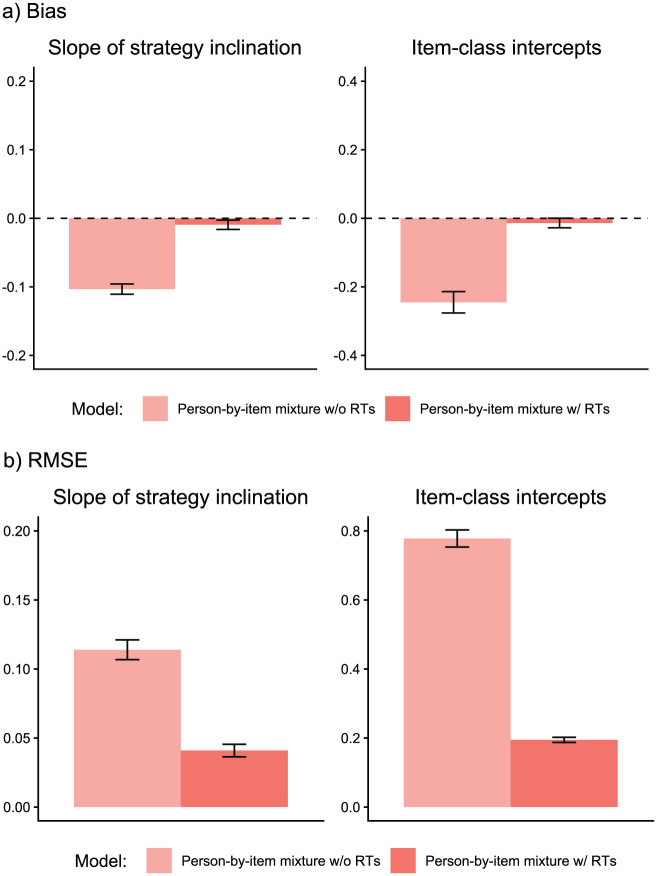
Parameter Recovery Study: Recovery of Latent Response Model Parameters. *Note.* Values reflect the mean bias (Panel a) or root mean square error (RMSE; Panel b) of estimated parameters across replications. Error bars represent the standard error of the mean. RT = response time.

**Figure 6.**
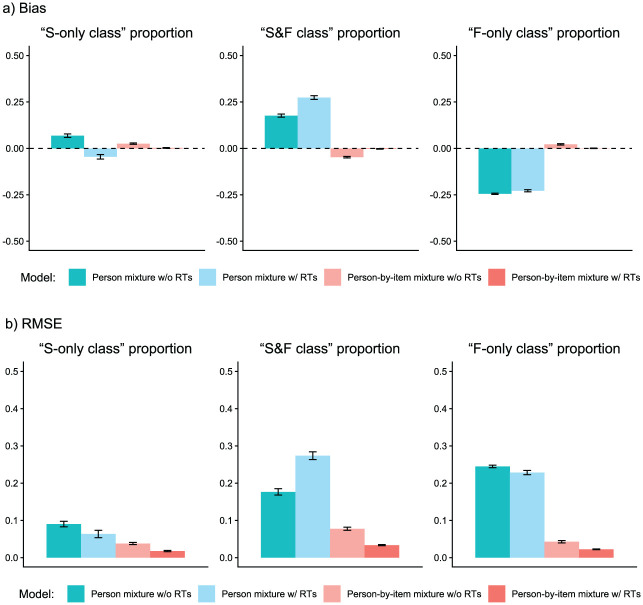
Parameter Recovery Study: Recovery of Class Proportions. *Note.* Values reflect the mean bias (Panel a) or root mean square error (RMSE; Panel b) of estimated parameters across replications. Class proportions are aggregated proportions across items. Error bars represent the standard error of the mean. RT = response time.

**Figure 7.**
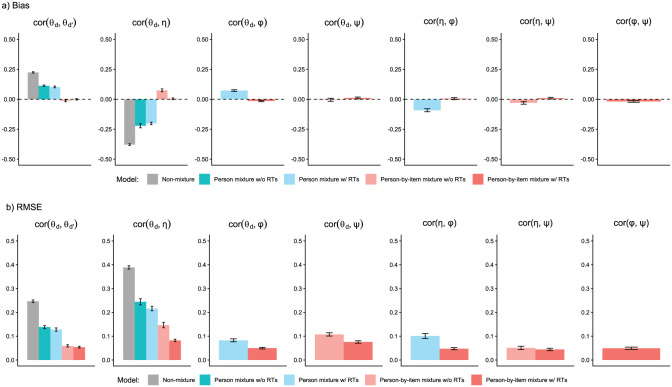
Parameter Recovery Study: Recovery of Latent Correlations. *Note.* Values reflect the mean bias (Panel a) or root mean square error (RMSE; Panel b) of estimated parameters across replications. Results for substantive traits are aggregated across the three substantive traits. Error bars represent the standard error of the mean. $\theta_{d}$ = substantive trait d ; η = faking; φ = speed; ψ = strategy inclination; RT = response time.

**Figure 8.**
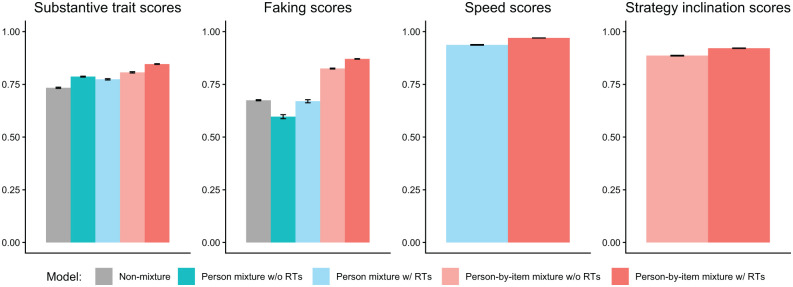
Parameter Recovery Study: Recovery of Person Parameters. *Note.* Values reflect the mean correlations (using Fisher’s *z*-transformation) between estimated and true parameters across replications. Results for substantive traits are aggregated across the three substantive traits. Error bars represent the standard error of the mean.

We analyzed parameter recovery based on converged models. In particular, we looked at bias to examine systematic over- or underestimation of parameters as well as at root mean square error (RMSE) to investigate estimation accuracy. For the recovery of person parameters, we considered the correlation between estimated and true parameters. Results are displayed in Figures 3–8. Overall, the data-generating person-by-item mixture model with RTs estimated parameters with negligible bias. Only for item-category intercepts, the model yielded slightly negatively biased estimates (corresponding to an average underestimation of 5-10% compared to the data-generating values). In contrast, all of the other models produced biased estimates for most of the model parameters. Regarding RMSE, too, the less complex models yielded for all parameters worse results than the person-by-item mixture model with RTs. Even though such a result pattern can in principle be expected since the data were generated based on a person-by-item mixture population, the results do indicate that conclusions drawn from models that do not capture the full data-generating process are indeed considerably less accurate. For instance, RMSE for RT model parameters (Figure 4b) and latent response model parameters (Figure 5b) was much larger in the corresponding person mixture model and person-by-item mixture model without RTs than in the full model. In addition, whereas both person-by-item mixture models yielded unbiased and fairly accurate estimates of class proportions, both person mixture models severely overestimated the overall proportion of the “S&F class” and underestimated the “F-only class” proportion (Figure 6). The person mixture model without RTs additionally overestimated the proportion of the “S-only class.” Regarding person parameters, the less complex models also yielded poorer estimates than the data-generating person-by-item mixture model with RTs (Figure 8). Effects were most pronounced for faking scores, where correlations between estimated and true person parameters differed considerably between models. For substantive trait, speed, and strategy inclination scores, the person-by-item mixture model with RTs yielded the highest correlations as well, but the less complex models did not perform much worse.

Furthermore, we examined the mixture models’ classification accuracy based on a modal class assignment rule (i.e., assignment to the class with the highest posterior class probability; Dias & Vermunt, 2008). We hereby looked at item-level hit rates, which indicate percentages of correct class assignments of individual item responses. The person-by-item mixture model with RTs yielded a mean hit rate of 63.3%, whereas the person-by-item mixture model without RTs only afforded a mean hit rate of 58.5%. Classification accuracy in the person mixture model with RTs (hit rate: 51.0%) and without RTs (hit rate: 51.7%) was even lower, which is conceivable given that a person’s class membership was not constant in the data generation. The size of the observed hit rates will be put into context in the Discussion section.

## Empirical Demonstration

To empirically demonstrate the proposed model, we analyzed data from an actual high-stakes job application context. Along with technical aspects like convergence and model fit in empirical settings, the empirical demonstration allowed investigating the consistency of test-takers’ response strategy use across items, class proportions on the level of items and the entire test, as well as RT differences associated with the three response strategies.

### Dataset

The data for the empirical demonstration were made available by a Germany-based testing company that develops psychological measurement tools for personnel selection. The dataset included *N* = 1,824 test-takers who had taken a personality test as part of their application for a police officer traineeship at a German police department between 2023 and 2024.^4^ The sample comprised 70.0% male and 30.0% female test-takers with a mean age of *M* = 21.02 years (*SD* = 4.61, *range* = [15, 39]). Along with item responses, the dataset contained item-level RTs (measured down to milliseconds). Exact timing of item-level response latencies was possible because every item had been presented on a separate questionnaire page.

For our empirical demonstration, we modeled item responses and RTs from three substantive trait scales available in the dataset. These were a scale of Emotional Stability (measured with 12 items; Cronbach’s α=.74 , McDonald’s ω=.73 ), a scale of Extraversion (9 items; α=.67 , ω=.65 ), and a scale of Conscientiousness (10 items; α=.77 , ω=.79 ). Across the substantive trait scales, items appeared in a random order. Responses were given on a 7-point Likert scale (0 = *does not apply at all* to 6 = *applies fully*).

### Pilot Study to Assess Desirability Values

We set scoring weights of substantive trait dimensions to $\left(\begin{array}{c} 0\;1\;2\;3\;4\;5\;6 \end{array}\right)$ and scoring weights of the faking dimension to values representing the social desirability of the items’ response categories with regard to the job application context at hand. These desirability values were collected in a pilot study (details are provided in Seitz, Alagöz, & Meiser, 2025, Pilot Study 2; https://osf.io/crmv4/), in which participants rated the desirability of every category of every item regarding the application for a police officer traineeship (see also Kuncel & Tellegen, 2009). Participants should thereby take the perspective of a person currently applying for such a job and rate desirability accordingly. The resulting mean desirability ratings then served as scoring weights of faking (see Figure 2 for three exemplary items). To achieve a common metric of scoring weights across latent dimensions, we linearly transformed the ratings to a possible range from 0 to 6.

### Results of the Empirical Demonstration

Like in the parameter recovery study, we fitted the person-by-item mixture model both with and without RTs as well as the person mixture model with and without RTs. We also fitted the three non-mixture models representing the measurement models of three modeled response strategies (Equations 1 to 3), that is, an MNRM, an MGPCM, and a unidimensional NRM with specified scoring weights of faking. We again used JAGS via the R environment for model estimation. In the empirical analysis, we estimated each model by running 12 parallel MCMC chains with 15,000 iterations following a 5000-iteration burnin phase.

#### Model Convergence and Model Fit

We checked model convergence based on $\hat{R}$ . For the person-by-item mixture model with RTs, all model parameters had $\hat{R}$ values below 1.1. A visual inspection of trace plots also showed well-mixed MCMC chains of the different model parameters. However, both the person-by-item mixture model without RTs and the person mixture model with RTs did not converge with all $\hat{R}$ values below 1.1, which is in line with the reduced convergence rates of these two models in the parameter recovery study. For the person-by-item mixture model without RTs, the non-convergence was mainly due to several item-class intercepts showing poorly mixed trace plots. For the person mixture model with RTs, the lack of convergence mainly stemmed from single item time intensities failing to converge. In contrast, the person mixture model without RTs as well as the three non-mixture models did converge with all $\hat{R}$ values below 1.1. In the following, we report results for the converged models only.

Table 1 shows model fit indices. The person-by-item mixture model with RTs yielded by far the highest log-likelihood.^5^ Crucially, this model was also selected by the widely applicable information criterion (WAIC; Watanabe, 2010) and the leave-one-out information criterion (LOOIC; Vehtari et al., 2017), indicating that the person-by-item mixture model with RTs yielded a better compromise between fit and parsimony than the other models. To quantify absolute fit, we applied posterior predictive model checking (PPMC; Sinharay et al., 2006), which is a technique that entails simulating data from the posterior distribution of model parameters and evaluating how the simulated data aligns with the observed data. Regarding item responses, we considered the standardized root mean square residual (SRMR), which indicates the misfit between model-implied and observed item intercorrelations. Compared to the other models, the SRMR of the person-by-item mixture model with RTs was smallest (.054), indicating that this model fit the item responses best. Regarding item RTs, we considered posterior predictive *p*-values (PPP) with respect to the discrepancy between model-implied and observed item means of log-RTs. Across items, PPPs ranged from .182 to .554 with a mean of .382, indicating that the RTs predicted by the model were not systematically higher or lower than the empirical RTs.^6^

**Table 1.** Empirical Demonstration: Model Fit Indices of Converged Models.

		Information criterion		PPMC	
Model	LL	WAIC	LOOIC	SRMR	RT-PPP
Non-mixture model (NRM)	−77,966.4	157,317.9	158,261.5	.142	
Non-mixture model (MGPCM)	−71,507.7	146,724.0	149,050.3	.101	
Non-mixture model (MNRM)	−68,981.6	142,372.6	144,994.4	.064	
Person mixture model w/o RTs	−67,561.1	139,844.2	142,550.6	.062	
**Person-by-item mixture model w/ RTs**	**−56,557.8** **(−61,785.7)**	**130,162.4** **(154,628.0)**	**132,862.1** **(157,201.5)**	**.054**	**.382** **[.182, .554]**

*Note.* *N* = 1,824. LL, WAIC, and LOOIC values outside round brackets were calculated with respect to item responses only, values in brackets with respect to the joint data of item responses and item response times (RT). LL = log-likelihood; WAIC = widely applicable information criterion; LOOIC = leave-one-out information criterion; PPMC = posterior predictive model checking; SRMR = standardized root mean square residual (based on PPMC); RT-PPP = mean posterior predictive *p*-value (range in square brackets) with respect to the log-RT item means; NRM = unidimensional nominal response model with specified scoring weights of faking; MGPCM = multidimensional generalized partial credit model; MNRM = multidimensional nominal response model. The best-fitting model is printed in bold.

Table 2 contains the latent correlations between dimensions estimated in the person-by-item mixture model with RTs. Most correlations were estimated not large in size, but different credibly from 0. An interpretation of the observed latent correlations is provided in the Discussion section.

**Table 2.** Empirical Demonstration: Estimated Latent Correlations

	$\theta_{1}$	$\theta_{2}$	$\theta_{3}$	η	φ	ψ
Emotional Stability ( $\theta_{1}$ )	1					
Extraversion ( $\theta_{2}$ )	.20 [.13, .27]	1				
Conscientiousness ( $\theta_{3}$ )	.24 [.17, .31]	.36 [.29, .43]	1			
Faking ( η )	−.23 [–.33, –.13]	−.01 [–.12, .11]	−.24 [–.35, –.14]	1		
Speed ( φ )	.11 [.06, .17]	.09 [.03, .15]	.08 [.02, .13]	.01 [–.07, .09]	1	
Strategy inclination ( ψ )	−.06 [–.13, .02]	.14 [.06, .21]	−.31 [–.38, –.24]	.32 [.22, .41]	−.00 [–.05, .05]	1

*Note.* *N* = 1,824. Values reflect the estimated latent correlations (95% credible intervals in brackets) in the person-by-item mixture model with response times.

#### Class Proportions and Class-Specific Item Response Distributions

With an estimated strategy inclination slope of $\hat{\nu_{\psi }}=1.05$ (95% credible interval (CrI): [1.00, 1.11]) and considerable variation in estimated item-class intercepts, the latent response model of the person-by-item mixture model with RTs implied class memberships that differed between both persons and items. Based on the modal assignment rule, 632 out of the 1,824 test-takers (34.6%) were classified into all three classes at least once across the items of the test, 561 test-takers (30.8%) were classified into two classes (“S-only”/“S&F”: 0; “S-only”/“F-only”: 245; “S&F”/“F-only”: 316), and 631 test-takers (34.6%) were constantly classified into one class (“S-only”: 614; “S&F”: 0; “F-only”: 17). This indicates that, while a constant class membership variable would have been sufficient for a considerable number of test-takers, allowing response strategy use to vary between items was empirically justified for the majority of test-takers.

Across all items, the person-by-item mixture model with RTs estimated that 48.6% (95% CrI: [47.0%, 50.1%]) of individual item responses stemmed from the “S-only class,” whereas 25.9% (95% CrI: [24.5%, 27.4%]) stemmed from the “S&F class” and 25.5% (95% CrI: [24.7%, 26.2%]) stemmed from the “F-only class.” The person mixture model without RTs estimated a similar class proportion for the “S-only class” (48.7%, 95% CrI: [44.7%, 52.7%]), however, it yielded a considerably larger “S&F class” proportion (47.0%, 95% CrI: [43.2%, 51.0%]) and a considerably smaller “F-only class” proportion (4.2%, 95% CrI: [3.2%, 5.3%]). Note that this pattern is in line with the parameter recovery results above, where overall proportions of the “S&F class” and “F-only class” were strongly biased in person mixture models.

**Figure 9.**
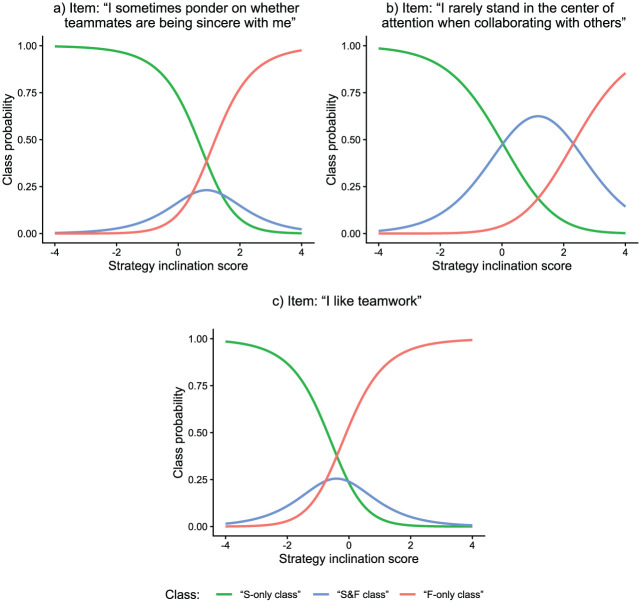
Empirical Demonstration: Latent Response Functions of Three Items. *Note.* Response functions are based on latent response model estimates from the person-by-item mixture model with response times. Item a had the largest “S-only class” proportion among all test items, item b the largest “S&F class” proportion, and item c the largest “F-only class” proportion.

Class proportions in the proposed model could also be computed item-specifically. Results showed that class proportions varied substantially from item to item. “S-only class” proportions ranged from 28.9% to 65.6% across items, “S&F class” proportions ranged from 9.3% to 44.2%, and “F-only class” proportions ranged from 8.0% to 51.6%. These estimates indicate that some items were more prone than other items to the different response strategies. At the same time, however, there were no items where all responses were based on the same strategy for all test-takers. Figure 9 displays class probabilities as a function of strategy inclination scores for the three items of the test with the largest estimated proportion of the “S-only class,”“S&F class,” and “F-only class,” respectively.

**Figure 10.**
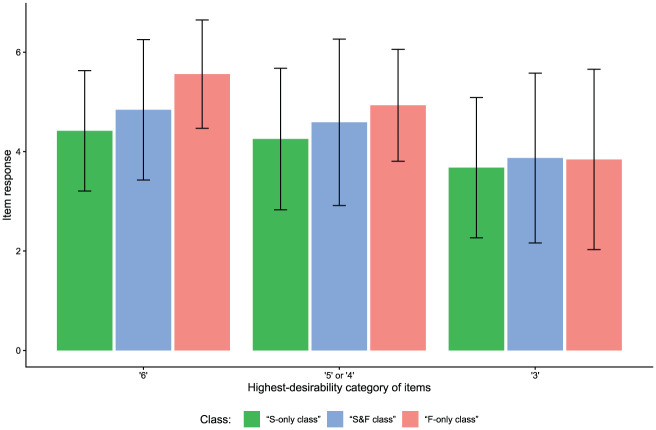
Empirical Demonstration: Class-Specific Mean Item Responses for Items With Different Desirability Characteristics. *Note.* Values reflect the mean item response for items with different highest-desirability response categories, split by the class test-takers were assigned to by the person-by-item mixture model with response times. Error bars represent the standard deviation.

Apart from different class sizes across items, the classes also differed in their response distributions depending on the items’ desirability characteristics (see Figure 10). For items at which the highest response category was most desirable (cf. Figure 2a), mean item responses were highest in the “F-only class” and lowest in the “S-only class.” This effect, though slightly less pronounced, also emerged for items having their category of highest desirability above the scale midpoint but not at the extreme (cf. Figure 2b). However, for items with a highest-desirability category at the scale midpoint (cf. Figure 2c), there were no considerable mean differences in item responses between classes.

#### Response Time Results

The person-by-item mixture model with RTs also yielded pronounced class differences concerning RTs. Across items, the mean “S-only class” time intensity was $\bar{\hat{\delta_{\cdot (S)}}}=1.72$ , whereas the mean “F-only class” time intensity was $\bar{\hat{\delta_{\cdot (F)}}}=1.53$ . This difference of 0.19 units on the log-RT scale was credibly different from 0 (95% CrI: [0.18, 0.20]). At the same time, the estimated “S&F class” proportionality constant was $\hat{\lambda }=0.23$ (95% CrI: [0.22, 0.24]), which was also substantially above its lower bound of 0. With time intensities of the “S&F class” being a linear function of “S-only class” and “F-only class” time intensities as well as the proportionality constant, the parameter estimates implied a mean “S&F class” time intensity of 2.08. On the level of raw item RTs, these estimates corresponded to median RTs of Mdn=5.86 seconds ( MAD=2.38) for responses classified into the “S-only class,” Mdn=6.50 seconds ( MAD=2.89) for “S&F class” responses, and Mdn=4.74 seconds ( MAD=2.07) for “F-only class” responses.

## General Discussion

In the current work, we presented a mixture IRT model that allows researchers and practitioners to account for, identify, and investigate different faking-related response strategies. The proposed model assumes that item responses of each test-taker are mixtures of three latent classes (pure substantive trait responding, response editing in the direction of desirability, and pure faking), and entails an RT-based latent response model that classifies item responses on the person-by-item level. RTs are modeled as indicators of class membership. That is, instead of being class membership predictors, RTs are reflections of strategy use and facilitate class separation by their relative typicality for the respective class. The model hence falls into the category of independent latent class IRT models (Nagy & Ulitzsch, 2022).

From a psychometric point of view, the presented person-by-item mixture modeling approach can flexibly account for heterogeneity in faking and thus constitutes an important extension over approaches assuming a constant faking strategy for each test-taker throughout the test or even a single measurement model for all test-takers. From a substantive research perspective, the proposed model can be used to study the response process behind faking in a sophisticated way. For example, differences in estimated item time intensity parameters reflect differences in RT distributions associated with the different response strategies, estimated item-class intercepts reveal which items are especially prone to a particular response strategy, and estimated latent correlations between substantive traits and faking and/or strategy inclination indicate how substantive person attributes are related to different faking tendencies. From an applied testing perspective, the model aids in ensuring the validity of inferences about test-takers, as substantive trait scores are estimated by accounting for potential switches between response strategies over the course of the assessment.

### Summary and Discussion of Results

#### Parameter Recovery

We examined the proposed model in a parameter recovery study with datasets representative of high-stakes assessment data. Along with examining parameter recovery of the proposed model, this study also allowed investigating the consequences of not modeling RTs as well as of assuming a constant response strategy per person or across the entire sample. In general, the proposed person-by-time mixture model with RTs exhibited good parameter recovery in terms of negligible bias and decent accuracy considering the not too large sample size in our parameter recovery study. Also, it was found that models that lack components of the data-generating process, which was based on the results of the empirical demonstration, indeed produce systematically biased and much less accurate parameter estimates than the proposed model. Several other findings stand out and are worth discussing.

First, whereas the person-by-item mixture model with RTs converged in almost all replications (98%), the person-by-item mixture model without RTs converged in only 74% of the replications. Non-convergence of the person-by-item mixture model without RTs was mainly due to several item-class intercepts in the latent response model component failing to converge. This is evidence that RTs have a facilitating effect on the estimation of the presented person-by-item mixture model despite not being direct predictors of class membership (see Nagy & Ulitzsch, 2022). Along with improving convergence, the results also suggest that modeling RTs has a positive effect on the accuracy of parameter estimation. In our parameter recovery study, the size of the effect varied between the different model parameters but was most pronounced for the latent response model parameters.

Second, the recovery of most model parameters differed substantially between person-by-item and person mixture models, with person mixture models yielding biased and less accurate estimates. This is not too surprising considering class membership varied both between persons and items in the data generation. However, it is important to note that person mixture models indeed lead to biased conclusions in data situations in which test-takers employ different response strategies over the course of the test (as was the case in our empirical demonstration). Particularly, class proportions estimated in person mixture models seem to be strongly biased, with the proportion of the “S&F class” systematically overestimated and the proportion of the “F-only class” systematically underestimated.^7^

Third, the parameter recovery study showed for the proposed model a rather low classification accuracy compared to other recent confirmatory mixture modeling approaches (e.g., Alagöz & Meiser, 2024; Seitz, Alagöz, & Meiser, 2025). Note, however, that the metric considered in the current article were hit rates on the item level (i.e., percentages of correct class assignments on the level of individual item responses), whereas articles studying classification accuracy of person mixture models considered hit rates on the level of test-takers. Taking into account that the information for assigning single item responses to latent classes is very sparse and naturally less reliable than the information for classifying whole response vectors, it is unsurprising that the obtained hit rates were not as high as the hit rates found in articles on person mixture models. Nevertheless, considering the size of the observed hit rates in the parameter recovery study, it is important to emphasize that response strategy classifications of individual item responses should be interpreted with caution. Because of the uncertainty associated with single classifications, it is arguably more advisable to look for general trends regarding the model-implied class memberships, be it for a particular test-taker or for a particular item.

#### Empirical Results

Seitz, Alagöz, and Meiser (2025) provided evidence for the prevalence of the three modeled response strategies on the person level. The empirical demonstration of the current article allowed investigating the consistency of response strategy use over the course of the assessment. As described above, the estimated parameters in the latent response model component implied class memberships that varied between persons and, crucially, within persons also between items. This empirically justifies the use of the more complex person-by-item mixture modeling approach, which is nevertheless flexible enough to yield a constant class membership for a person whose response pattern suggests that. This flexibility of the model indeed yielded an interesting classification pattern regarding how consistently test-takers used the three response strategies. Namely, if test-takers used an “S-only” strategy, they usually did so across all items, whereas test-takers engaging in faking mostly switched between an “S&F” and “F-only” strategy.

Regarding the overall class proportions, the model estimated that about one-half of individual item responses stemmed from the “S-only class,” whereas about one-quarter each stemmed from the “S&F class” and the “F-only class.” That is, according to the model, around half of the responses in total were honest in terms of being solely influenced by substantive traits, whereas the other half were at least partially influenced by faking. These proportions are well in line with the proportions Brown and Böckenholt (2022) found in their model including only two classes (49% honest vs. 51% faked). However, our results suggest that some responses influenced by faking can still contain information on substantive traits and hence do not need to be discarded for the estimation of substantive trait scores.

With respect to the latent correlations between dimensions, the model’s estimates were generally not large in size, but most were credibly different from 0. For instance, the substantive traits were estimated to be moderately positively correlated, which is in line with research on associations between the Big Five personality factors (e.g., van der Linden et al., 2010), but were not unreasonably inflated as is typically observed in high-stakes assessments when faking is not statistically accounted for (e.g., Schmit & Ryan, 1993; Seitz, Spengler, & Meiser, 2025). At the same time, there were non-negligible latent correlations of substantive traits with faking and strategy inclination. Conscientiousness, for instance, was estimated to be moderately negatively correlated with both faking (–.24) and strategy inclination (–.31). However, the latent correlations were not so high that one would have to assume that faking and strategy inclination absorb a disproportionately large amount of substantive personality variance. Furthermore, note the estimated latent correlation between faking and strategy inclination. This correlation of .32 indicates a positive association between aligning responses with desirability and using a pronounced self-presentation strategy; however, it also speaks against a full overlap of the two dimensions. Hence, to sum up, the model’s estimated latent correlations provide validity evidence regarding the meaning and non-redundancy of the modeled latent dimensions.

Another interesting empirical finding concerns the model’s RT results, indicating that pure faking is faster than pure substantive trait responding and that a combination of the two processes takes longest. These findings could reconcile some of the conflicting results in the literature regarding the effect of faking on RTs. In particular, our findings suggest that the question of whether faking increases or decreases RTs cannot be answered straightforwardly, but that it depends on the response process underlying a response that involves faking. If test-takers retrieve an honest response before they edit this response according to desirability (i.e., “S&F class”; cf. the theory by Walczyk et al., 2003), the results suggest that this goes along with increased RTs (Fine & Pirak, 2016; Holtgraves, 2004; Walczyk et al., 2003). Such a response set corresponds to the response set triggered by the experimental condition in the study by Holtgraves (2004), who induced faking along with honest responding through a subtle desirability manipulation. If, however, test-takers bypass the retrieval of an honest response and instead just provide a desirable answer (i.e., “F-only class”; cf. the theory by Holden, 1995), the results suggest that this reduces RTs. Such a response set is analogous to response sets induced through mere instructed-faking conditions, in which subjects are simply asked to fake without referring to their true personality (Holden, 1995; Holden et al., 1992; Hsu et al., 1989). Taking this into account, the model’s RT results imply a limited potential of simple (i.e., non-model-based) faking detection methods that perform faking classifications based on a single RT threshold (Fine & Pirak, 2016).

### Limitations and Future Research Directions

Some limitations and directions for future research need to be mentioned. One aspect concerns the model’s assumptions, which need to be kept in mind when applying the model and interpreting its parameter estimates: First, as is also the case in non-mixture applications of the MNRM when modeling faking, the specification of scoring weights of faking is constant across persons. That is, it is implicitly assumed that test-takers do not differ systematically in the mere perception of social desirability. A simulation study on the non-mixture version of the model might have shown that the model is fairly robust when test-takers perceive desirability differently (Kleinbub & Seitz, 2025), but it remains to be investigated how this transfers to the model extension of the current article. Second, in order for RTs to have a facilitating effect on model estimation and class separation, there should be differences in RT distributions between classes. With RT distributions becoming less distinct, one can expect the facilitating effect of modeling RTs shown in the parameter recovery study to weaken and eventually disappear (Pokropek, 2016; Ulitzsch et al., 2024). Third, along with separable RT distributions, item response distributions should also exhibit good between-class separability. Future studies can explore which questionnaire characteristics are required to achieve this (cf. Uglanova et al., 2025). Fourth, completion speed is assumed to be constant across the test. This stationarity assumption (van der Linden, 2007), however, is likely to be violated when test-takers become exhausted at later parts of the assessment or become more acquainted with the questionnaire format.

Another aspect of limitations and future research directions concerns the current parameterizations of some of the model components. For instance, the latent response model has been parametrized as a PCM, which assumes the classes to be ordinal. For a more general formulation, the latent response model could in principle also be implemented as an NRM, which would relax the ordinality assumption. However, in initial simulation runs in the model development phase, we found this increased estimation complexity to pose a challenge to model convergence. Different parameter restrictions would probably be necessary in this case. Likewise, future studies could try to estimate time intensities of the “S&F class” in a less restrictive way. Based on theoretical considerations, we constrained “S&F class” time intensities to be a function of “S-only class” and “F-only class” time intensities and restricted the proportionality constant λ to be positive. The proportionality constant indeed turned out to be substantially larger than 0 in the empirical demonstration, but an unconstrained estimation of “S&F class” time intensities would nonetheless be interesting. Moreover, a different parameterization of the “F-only class” could be tested, namely one with a model of stochastic independence. In such a model, faking would only be captured by item-category intercepts, with no variance explained by a common factor such that all variation in this class would be unsystematic.

A further alternative way of parameterizing the model would also be to implement item-level predictors of class membership and/or RTs. This could be achieved by restricting item-class intercepts and/or item time intensities through linear combinations of variables that describe the items, such as item keying, item length, item position, or item content features (see Ulitzsch, Yildirim-Erbasli, et al., 2022). Researchers could use such a parametrization to study item characteristics that make certain response strategies especially likely or have a systematic effect on response latencies. If, for instance, particular item characteristics were found to strongly predict “F-only class” membership, this would be a valuable piece of information for test construction, as it could help to develop instruments that are less susceptible to faking in the first place.

In addition, it would be important to conduct validation analyses in future studies. From the perspective of applied measurement, it would be particularly relevant to further investigate the validity of the model’s adjustments of substantive trait score estimates because substantive trait scores are the parameters of primary interest in contexts where test-takers are ranked and selected based on their test scores. In this regard, future research should study whether the adjustments of substantive trait score estimates increase correlations with faking-resistant measures of personality. Ultimately, it would also be interesting to see the effects of the adjustments on the prediction of job-relevant performance outcomes.

Finally, practical issues associated with the presented method of modeling faking are to be mentioned. In particular, the presented approach comes with the limitation of requiring a lot of resources in the form of computation power and time, as fitting the model can demand a substantial amount of working memory and take quite some time to achieve convergence. The actual estimation time will ultimately depend on sample size, test length, and the available computer. In the case of our parameter recovery study, a single estimation of the model took around twelve hours on a high-performance computer. This can be a major obstacle for applications of the model in practice. However, estimation time can be drastically reduced in an applied measurement context where only person parameters of new incoming test-takers need to be estimated. Here, one can estimate model parameters once in a sufficiently large calibration sample and then treat person-invariant parameters as fixed for the estimation of person parameters of additional test-takers. Computation time will in this case reduce to just a few seconds.

## Conclusion

In conclusion, the presented RT-based person-by-item mixture model constitutes an appealing approach to modeling faking in high-stakes personality testings. As opposed to most other faking models, it accounts for switches between different faking-related response strategies over the course of the assessment. It thereby makes use of additional behavioral data by modeling strategy-specific RT distributions, thus allowing for a sophisticated investigation of the response process associated with faking. Future research can examine different parametrizations of the model and conduct validation analyses of particular model parameters.

## Supplemental Material

sj-docx-1-epm-10.1177_00131644261422169 – Supplemental material for Faking in High-Stakes Personality Assessments: A Response-Time-Based Latent Response Mixture Modeling Approach Supplemental material, sj-docx-1-epm-10.1177_00131644261422169 for Faking in High-Stakes Personality Assessments: A Response-Time-Based Latent Response Mixture Modeling Approach by Timo Seitz and Esther Ulitzsch in Educational and Psychological Measurement

## Appendix

### Pre-Study to Assess Response Time Behavior

As discussed in the Main Text, the literature is split concerning the overall effect of faking on response times (RT), with some studies finding shorter RTs (e.g., Holden, 1995; Holden et al., 1992; Hsu et al., 1989) and other studies finding longer RTs associated with faking (e.g., Fine & Pirak, 2016; Holtgraves, 2004; Walczyk et al., 2003). However, the studies differ in the operationalization of “faking.” Some used direct instructed-faking manipulations (e.g., Holden, 1995; Holden et al., 1992; Hsu et al., 1989), others used manipulations that should elevate desirability concerns in a more subtle way, for instance, by telling participants that their responses would be used to create a personality profile of them (Holtgraves, 2004).

To facilitate our understanding of RT effects associated with the three response strategies modeled in this article, and to empirically corroborate our assumption of increased RTs in the “S&F class,” we conducted a pre-study using an experimental design. Therefore, we collected data from *N* = 72 participants (gender: 52.8% female, 47.2% male; age: *M* = 22.83 years, *SD* = 3.18, *range* = [19, 35]) on the online platform *SoSci Survey* (https://www.soscisurvey.de/) and instructed all of them to respond to a set of personality items in three conditions. In one condition (analogous to the “S-only class” in our model), participants should be fully honest in their responses, that is, they should base their responses on their true personality. In a second condition (analogous to the “S&F class”), participants should in principle also be honest but embellish their responses in the direction of social desirability. In a third condition (analogous to the “F-only class”), participants should base their responses solely on the items’ desirability, without referring to their actual personality. The order of conditions was counterbalanced between participants. The items were the actual items of the test used in the high-stakes assessment from our empirical demonstration. Responses were given on a 7-point Likert scale (1 = *very low agreement* to 7 = *very high agreement*). Like in the actual assessment from our empirical demonstration, each item appeared on a separate questionnaire page, which allowed exact measurement of RTs on the item level. The order of items within conditions was also counterbalanced between participants. More information can be accessed at https://osf.io/crmv4/.

For analysis, we ran a series of multilevel regression models using the R packages *lme4* (Bates et al., 2015) and *lmerTest* (Kuznetsova et al., 2017), and set the significance level to α=.05 . We excluded RTs below 1 second and above 30 seconds, and log-transformed RTs to approximate a normal distribution. With individual observations nested in persons as well as items, we specified random intercepts for persons and random intercepts for items (i.e., cross-classified random effects). Table A1 shows the results of the fitted multilevel models. In the model without person and item covariates, the “S&F class” condition was associated with significantly longer RTs compared to the “S-only class” condition, whereas the “F-only class” was associated with significantly shorter RTs compared to the “S-only class” condition. These effects remained significant when adding person (gender, age) and item covariates (item keying, number of characters per item). Descriptively, the effects of the conditions were rather small, with median RTs of Mdn=3.87 seconds ( MAD=1.68 ) in the “S-only class” condition, Mdn=4.32 seconds ( MAD=2.36 ) in the “S&F class” condition, and Mdn=3.67 seconds ( MAD=1.79 ) in the “F-only class” condition. A repeated-measures analysis of variance (ANOVA) yielded an effect size of $\eta_{G}^{2}=.051$ ( F(2,142)=19.69,p<.001 ).

**Table A1.** Parameter Estimates, Standard Errors, and Model Fit Indices of the Multilevel Models of the Pre-Study.

Effect	Parameter estimate (standard error)				
	Model 0	Model 1	Model 2	Model 3	Model 4
*Fixed effects:*					
*(Intercept)*	1.40 (0.04)***	1.38 (0.04)***	1.37 (0.05)***	1.34 (0.04)***	1.33 (0.05)***
Conditions:					
“S&F class” condition		0.12 (0.01)***	0.12 (0.01)***	0.12 (0.01)***	0.12 (0.01)***
“F-only class” condition		−0.04 (0.01)***	−0.04 (0.01)***	−0.04 (0.01)***	−0.04 (0.01)***
Person covariates:					
Gender			0.02 (0.06)		0.02 (0.06)
Age			0.05 (0.03)^†^		0.05 (0.03)^†^
Item covariates:					
Keying				0.10 (0.03)**	0.10 (0.03)**
Number of characters				0.01 (0.00) ***	0.01 (0.00) ***
*Random effects:*					
Level 1 residual variance	0.20	0.20	0.20	0.20	0.20
Level 2 residual variance due to persons	0.07	0.07	0.07	0.07	0.07
Level 2 residual variance due to items	0.02	0.02	0.02	0.01	0.01
*Model fit indices:*					
Log-likelihood	−6,608.3	−6,484.6	−6,483.1	−6,468.3	−6,466.8
Deviance	13,216.6	12,969.2	12,966.2	12,936.6	12,933.7
AIC	13,224.6	12,981.2	12,982.2	12,952.6	12,953.7
BIC	13,253.5	13,024.5	13,040.1	13,010.4	13,026.0

*Note. N* = 72 (10,209 observations). Condition (“S-only class” condition as reference), gender (male as reference), and item keying (positively keyed as reference) were dummy-coded; age was *z*-standardized; number of characters per item was centered. AIC = Akaike information criterion; BIC = Bayesian information criterion. *p*-values are two-tailed.

† *p* < .10, **p* < .05, ***p* < .01, ****p* < .001.

With these results, the pre-study provides evidence for existing RT differences between the different response strategies. In particular, RTs were longest when participants responded according to substantive traits *and* desirability, supporting the λ>0 constraint in our proposed model. Also, the multilevel analyses yielded substantial random intercept variances for both persons and items. These were not considerably reduced when adding person and item covariates, emphasizing the importance of having a person speed parameter and item-specific time intensity parameters in the proposed model.

## Notes

1. Note that, as with all latent variables, faking scores are a normative characterization of faking behavior (see Bolt & Meng, 2025). That is, negative faking scores do not indicate “faking bad” in the sense of socially *un*desirable responding, nor does a faking score of 0 indicate the absence of faking. Instead, faking scores should be interpreted as values on a latent continuum, where 0 is usually the latent mean that is set for model identification. Negative faking scores simply reflect a faking level that is below average, whereas the absence of faking is rather implied by a faking slope of 0, which is the case in the model’s “S-only class.”

2. To corroborate the assumption of longest RTs in the “S&F class,” we conducted a pre-study in which we assessed RT behavior associated with the three modeled response strategies (see Appendix). In line with theoretical considerations, RTs in the pre-study were indeed longest when participants responded according to substantive traits and desirability. Also, RTs were shorter when participants responded only according to desirability than when they responded only according to substantive traits.

3. Mixture models can be challenging to estimate because of multimodal likelihood functions (McLachlan & Peel, 2000), which make single MCMC chains susceptible to converging to local solutions. To alleviate this problem, we employed an estimation strategy that optimizes initial values before the actual estimation (cf. O’Hagan et al., 2012). In the parameter recovery study, this was done by running 12 (30 in the empirical demonstration) parallel chains (each with 2,000 burnin and 2,000 regular iterations (3,000+3,000 in the empirical demonstration)) with unsystematic initial values and taking the parameter estimates from the chain yielding the highest model likelihood as initial values for the actual model estimation. The actual estimation then featured the reported numbers of chains and iterations, with random noise added to the initial values of each chain to avoid that every chain started with the exact same set of values. Details can be retrieved at https://osf.io/crmv4/.

4. This dataset was also analyzed by Seitz, Alagöz, and Meiser (2025), but their analysis did not involve RTs and was limited to a person mixture model.

5. Note that likelihood-based comparisons (i.e., log-likelihood differences, WAIC, and LOOIC) between models accounting versus not accounting for RTs are only warranted if the likelihood is computed with respect to item responses only. If the likelihood is computed with respect to the full modeled data, likelihoods of the different models are not comparable because models not accounting for RTs only comprise item response data and models accounting for RTs additionally comprise item RT data.

6. In particular, item responses and item RTs were simulated for each MCMC iteration after the burnin phase using the respective model equation and sampled parameter values in the given iteration. Regarding item response data, the SRMR of a model was calculated by computing the root mean squared deviation between item intercorrelations in the simulated versus observed data per iteration before averaging across iterations. Regarding item RT data, item-wise PPPs were computed as percentages of simulated log-RT item means being greater than or equal to the corresponding observed item mean across iterations.

7. One should keep in mind that the level class proportion estimates refer to differs between person-by-item and person mixture models. Whereas estimates in person-by-item mixture models refer to class proportions on the level of individual item responses, estimates in person mixture models refer to class proportions on the level of test-takers. Class proportion parameters hence do not have the same conceptual meaning in the two types of mixture models.

## References

[bibr1-00131644261422169] Alagöz Ö. E. C. Meiser T . (2024). Investigating heterogeneity in response strategies: A mixture multidimensional IRTree approach. Educational and Psychological Measurement, 84(5), 957–993. 10.1177/00131644231206765 39318480 PMC11418595

[bibr2-00131644261422169] Bates D. Mächler M. Bolker B. Walker S. (2015). Fitting linear mixed-effects models using lme4. Journal of Statistical Software, 67(1), 1–48. 10.18637/jss.v067.i01

[bibr3-00131644261422169] Bock R. D. (1972). Estimating item parameters and latent ability when responses are scored in two or more nominal categories. Psychometrika, 37(1), 29–51. 10.1007/bf02291411

[bibr4-00131644261422169] Böckenholt U. (2014). Modeling motivated misreports to sensitive survey questions. Psychometrika, 79(3), 515–537. 10.1007/s11336-013-9390-9 24297438

[bibr5-00131644261422169] Bolt D. M. Meng L. (2025). IRT-based response style models and related methodology: Review and commentary. British Journal of Mathematical and Statistical Psychology. Advance online publication. 10.1111/bmsp.70006 40824188

[bibr6-00131644261422169] Brown A. Böckenholt U. (2022). Intermittent faking of personality profiles in high-stakes assessments: A grade of membership analysis. Psychological Methods, 27(5), 895–916. 10.1037/met0000295 35007104

[bibr7-00131644261422169] Denwood M. J. (2016). Runjags: An R package providing interface utilities, model templates, parallel computing methods and additional distributions for MCMC models in JAGS. Journal of Statistical Software, 71(9), 1–25. 10.18637/jss.v071.i09

[bibr8-00131644261422169] Dias J. G. Vermunt J. K. (2008). A bootstrap-based aggregate classifier for model-based clustering. Computational Statistics, 23(4), 643–659. 10.1007/s00180-007-0103-7

[bibr9-00131644261422169] Ellingson J. E. McFarland L. A. (2011). Understanding faking behavior through the lens of motivation: An application of VIE theory. Human Performance, 24(4), 322–337. 10.1080/08959285.2011.597477

[bibr10-00131644261422169] Falk C. F. Cai L. (2016). A flexible full-information approach to the modeling of response styles. Psychological Methods, 21(3), 328–347. 10.1037/met0000059 26641273

[bibr11-00131644261422169] Fine S. Pirak M. (2016). Faking fast and slow: Within-person response time latencies for measuring faking in personnel testing. Journal of Business and Psychology, 31(1), 51–64. 10.1007/s10869-015-9398-5

[bibr12-00131644261422169] Gelman A. Rubin D. B. (1992). Inference from iterative simulation using multiple sequences. Statistical Science, 7(4), 457–472. 10.1214/ss/1177011136

[bibr13-00131644261422169] Griffith R. L. Converse P. D. (2011). The rules of evidence and the prevalence of applicant faking. In Ziegler M. MacCann C. Roberts R. D. (Eds.), New perspectives on faking in personality assessment (pp. 34–52). Oxford University Press. 10.1093/acprof:oso/9780195387476.003.0018

[bibr14-00131644261422169] Hendy N. Krammer G. Schermer J. A. Biderman M. D. (2021). Using bifactor models to identify faking on Big Five questionnaires. International Journal of Selection and Assessment, 29(1), 81–99. 10.1111/ijsa.12316

[bibr15-00131644261422169] Holden R. R. (1995). Response latency detection of fakers on personnel tests. Canadian Journal of Behavioural Science, 27(3), 343–355. 10.1037/0008-400x.27.3.343

[bibr16-00131644261422169] Holden R. R. Kroner D. G. Fekken G. C. Popham S. M. (1992). A model of personality test item response dissimulation. Journal of Personality and Social Psychology, 63(2), 272–279. 10.1037/0022-3514.63.2.272

[bibr17-00131644261422169] Holtgraves T. (2004). Social desirability and self-reports: Testing models of socially desirable responding. Personality and Social Psychology Bulletin, 30(2), 161–172. 10.1177/0146167203259930 15030631

[bibr18-00131644261422169] Hsu L. M. Santelli J. Hsu J. R. (1989). Faking detection validity and incremental validity of response latencies to MMPI Subtle and Obvious items. Journal of Personality Assessment, 53(2), 278–295. 10.1207/s15327752jpa5302_6

[bibr19-00131644261422169] Hu J. Connelly B. S. (2021). Faking by actual applicants on personality tests: A meta-analysis of within-subjects studies. International Journal of Selection and Assessment, 29(3–4), 412–426. 10.1111/ijsa.12338

[bibr20-00131644261422169] Jasra A. Holmes C. C. Stephens D. A. (2005). Markov chain Monte Carlo methods and the label switching problem in Bayesian mixture modeling. Statistical Science, 20(1), 50–67. 10.1214/088342305000000016

[bibr21-00131644261422169] Klehe U.-C. Kleinmann M. Hartstein T. Melchers K. G. König C. J. Heslin P. A. Lievens F. (2012). Responding to personality tests in a selection context: The role of the ability to identify criteria and the ideal-employee factor. Human Performance, 25(4), 273–302. 10.1080/08959285.2012.703733

[bibr22-00131644261422169] Kleinbub J. D. Seitz T. (2025, September 29–October 1). Unmasking the faker: Heterogeneous perception of social desirability in context of the multidimensional nominal response model [Poster]. 17th Meeting of the Methods and Evaluation Division of the German Psychological Society (DGPs), Berlin, Germany.

[bibr23-00131644261422169] Kuncel N. R. Goldberg L. R. Kiger T. (2011). A plea for process in personality prevarication. Human Performance, 24(4), 373–378. 10.1080/08959285.2011.597476 PMC3182471 21966260

[bibr24-00131644261422169] Kuncel N. R. Tellegen A. (2009). A conceptual and empirical reexamination of the measurement of the social desirability of items: Implications for detecting desirable response style and scale development. Personnel Psychology, 62(2), 201–228. 10.1111/j.1744-6570.2009.01136.x

[bibr25-00131644261422169] Kuznetsova A. Brockhoff P. B. Christensen R. H. B. (2017). LmerTest package: Tests in linear mixed effects models. Journal of Statistical Software, 82(13), 1–26. 10.18637/jss.v082.i13

[bibr26-00131644261422169] Leng C. H. Huang H. Y. Yao G. (2020). A social desirability item response theory model: Retrieve-deceive-transfer. Psychometrika, 85(1), 56–74. 10.1007/s11336-019-09689-y 31677045

[bibr27-00131644261422169] Maris E. (1995). Psychometric latent response models. Psychometrika, 60(4), 523–547. 10.1007/bf02294327

[bibr28-00131644261422169] Masters G. N. (1982). A Rasch model for partial credit scoring. Psychometrika, 47(2), 149–174. 10.1007/bf02296272

[bibr29-00131644261422169] Mazar N. Amir O. Ariely D. (2008). The dishonesty of honest people: A theory of self-concept maintenance. Journal of Marketing Research, 45(6), 633–644. 10.1509/jmkr.45.6.633

[bibr30-00131644261422169] McLachlan G. Peel D. (2000). Finite mixture models. Wiley.

[bibr31-00131644261422169] Messick S. (1989). Meaning and values in test validation: The science and ethics of assessment. Educational Researcher, 18(2), 5–11. 10.3102/0013189x018002005

[bibr32-00131644261422169] Mueller-Hanson R. Heggestad E. D. Thornton G. C. (2003). Faking and selection: Considering the use of personality from select-in and select-out perspectives. Journal of Applied Psychology, 88(2), 348–355. 10.1037/0021-9010.88.2.348 12731719

[bibr33-00131644261422169] Muraki E. (1992). A generalized partial credit model: Application of an EM algorithm. Applied Psychological Measurement, 16(2), 159–176. 10.1177/014662169201600206

[bibr34-00131644261422169] Nagy G. Ulitzsch E. (2022). A multilevel mixture IRT framework for modeling response times as predictors or indicators of response engagement in IRT models. Educational and Psychological Measurement, 82(5), 845–879. 10.1177/00131644211045351 35989730 PMC9386881

[bibr35-00131644261422169] O’Hagan A. Murphy T. B. Gormley I. C. (2012). Computational aspects of fitting mixture models via the expectation–maximization algorithm. Computational Statistics & Data Analysis, 56(12), 3843–3864. 10.1016/j.csda.2012.05.011

[bibr36-00131644261422169] Plummer M. (2017). JAGS: Just another Gibbs sampler (version 4.3.2) [Computer software]. https://sourceforge.net/projects/mcmc-jags/

[bibr37-00131644261422169] Plummer M. Best N. Cowles K. Vines K. (2006). CODA: Convergence diagnosis and output analysis for MCMC. R News, 6(1), 7–11. https://www.r-project.org/doc/Rnews/Rnews_2006-1.pdf

[bibr38-00131644261422169] Pokropek A. (2016). Grade of membership response time model for detecting guessing behaviors. Journal of Educational and Behavioral Statistics, 41(3), 300–325. 10.3102/1076998616636618

[bibr39-00131644261422169] Poropat A. E. (2009). A meta-analysis of the five-factor model of personality and academic performance. Psychological Bulletin, 135(2), 322–338. 10.1037/a0014996 19254083

[bibr40-00131644261422169] Robie C. Brown D. J. Beaty J. C. (2007). Do people fake on personality inventories? A verbal protocol analysis. Journal of Business and Psychology, 21(4), 489–509. 10.1007/s10869-007-9038-9

[bibr41-00131644261422169] Röhner J. Schütz A. Ziegler M. (2025). Faking in self-report personality Scales: A qualitative analysis and taxonomy of the behaviors that constitute faking strategies. International Journal of Selection and Assessment, 33(1), Article e12513. 10.1111/ijsa.12513

[bibr42-00131644261422169] Sackett P. R. Walmsley P. T. (2014). Which personality attributes are most important in the workplace? Perspectives on Psychological Science, 9(5), 538–551. 10.1177/1745691614543972 26186756

[bibr43-00131644261422169] Schmit M. J. Ryan A. M. (1993). The Big Five in personnel selection: Factor structure in applicant and nonapplicant populations. Journal of Applied Psychology, 78(6), 966–974. 10.1037/0021-9010.78.6.966

[bibr44-00131644261422169] Seitz T. Alagöz Ö. E. C. Meiser T. (2025). Disentangling qualitatively different faking strategies in high-stakes personality assessments: A mixture extension of the multidimensional nominal response model. Educational and Psychological Measurement, 85(6), 1237–1277. 10.1177/00131644251341843 PMC12310618 40756699

[bibr45-00131644261422169] Seitz T. Spengler M. Meiser T. (2025). “What if applicants fake their responses?” Modeling faking and response styles in high-stakes assessments using the multidimensional nominal response model. Educational and Psychological Measurement, 85(4), 747–782. 10.1177/00131644241307560 39866184 PMC11755426

[bibr46-00131644261422169] Seitz T. Wetzel E. Hilbig B. E. Meiser T. (2024). Using the multidimensional nominal response model to model faking in questionnaire data: The importance of item desirability characteristics. Behavior Research Methods, 56(8), 8869–8896. 10.3758/s13428-024-02509-x 39304600 PMC11525249

[bibr47-00131644261422169] Sinharay S. Johnson M. S. Stern H. S. (2006). Posterior predictive assessment of item response theory models. Applied Psychological Measurement, 30(4), 298–321. 10.1177/0146621605285517

[bibr48-00131644261422169] Sudman S. Bradburn N. M. Schwarz N. (1996). Thinking about answers: The application of cognitive processes to survey methodology. Jossey-Bass.

[bibr49-00131644261422169] Takane Y. de Leeuw J. (1987). On the relationship between item response theory and factor analysis of discretized variables. Psychometrika, 52(3), 393–408. 10.1007/bf02294363

[bibr50-00131644261422169] Tett R. P. Simonet D. V. (2011). Faking in personality assessment: A “multisaturation” perspective on faking as performance. Human Performance, 24(4), 302–321. 10.1080/08959285.2011.597472

[bibr51-00131644261422169] Tourangeau R. Yan T. (2007). Sensitive questions in surveys. Psychological Bulletin, 133(5), 859–883. 10.1037/0033-2909.133.5.859 17723033

[bibr52-00131644261422169] Uglanova I. Nagy G. Ulitzsch E. (2025, February 20). A mixture IRT model for handling different types of careless respondents. PsyArXiv. 10.31219/osf.io/tgys3_v2

[bibr53-00131644261422169] Ulitzsch E. Nestler S. Lüdtke O. Nagy G. (2024). A screen-time-based mixture model for identifying and monitoring careless and insufficient effort responding in ecological momentary assessment data. Psychological Methods. Advance online publication. 10.1037/met0000636 38421768

[bibr54-00131644261422169] Ulitzsch E. Pohl S. Khorramdel L. Kroehne U. von Davier M. (2022). A response-time-based latent response mixture model for identifying and modeling careless and insufficient effort responding in survey data. Psychometrika, 87(2), 593–619. 10.1007/s11336-021-09817-7 34855118 PMC9166878

[bibr55-00131644261422169] Ulitzsch E. von Davier M. Pohl S. (2020). A hierarchical latent response model for inferences about examinee engagement in terms of guessing and item-level non-response. British Journal of Mathematical and Statistical Psychology, 73(S1), 83–112. 10.1111/bmsp.12188 31709521

[bibr56-00131644261422169] Ulitzsch E. Yildirim-Erbasli S. N. Gorgun G. Bulut O. (2022). An explanatory mixture IRT model for careless and insufficient effort responding in self-report measures. British Journal of Mathematical and Statistical Psychology, 75(3), 668–698. 10.1111/bmsp.12272 35730351

[bibr57-00131644261422169] van der Linden D. te Nijenhuis J. Bakker A. B . (2010). The general factor of personality: A meta-analysis of Big Five intercorrelations and a criterion-related validity study. Journal of Research in Personality, 44(3), 315–327. 10.1016/j.jrp.2010.03.003

[bibr58-00131644261422169] van der Linden W. J . (2006). A lognormal model for response times on test items. Journal of Educational and Behavioral Statistics, 31(2), 181–204. 10.3102/10769986031002181

[bibr59-00131644261422169] van der Linden W. J . (2007). A hierarchical framework for modeling speed and accuracy on test items. Psychometrika, 72(3), 287–308. 10.1007/s11336-006-1478-z

[bibr60-00131644261422169] Vehtari A. Gelman A. Gabry J. (2017). Practical Bayesian model evaluation using leave-one-out cross-validation and WAIC. Statistics and Computing, 27(5), 1413–1432. 10.1007/s11222-016-9696-4

[bibr61-00131644261422169] Venables W. N. Ripley B. D. (2002). Modern applied statistics with S (4th ed.). Springer.

[bibr62-00131644261422169] Walczyk J. J. Roper K. S. Seemann E. Humphrey A. M. (2003). Cognitive mechanisms underlying lying to questions: Response time as a cue to deception. Applied Cognitive Psychology, 17(7), 755–774. 10.1002/acp.914

[bibr63-00131644261422169] Watanabe S. (2010). Asymptotic equivalence of Bayes cross validation and widely applicable information criterion in singular learning theory. Journal of Machine Learning Research, 11, 3571–3594.

[bibr64-00131644261422169] Wolodzko T. (2023). extraDistr: Additional univariate and multivariate distributions (version 1.10.0) [Computer software]. https://cran.r-project.org/web/packages/extraDistr/index.html

[bibr65-00131644261422169] Youngflesh C. (2018). MCMCvis: Tools to visualize, manipulate, and summarize MCMC output. Journal of Open Source Software, 3(24), 640. 10.21105/joss.00640

[bibr66-00131644261422169] Zickar M. J. Gibby R. E. Robie C. (2004). Uncovering faking samples in applicant, incumbent, and experimental datasets: An application of mixed-model item response theory. Organizational Research Methods, 7(2), 168–190. 10.1177/1094428104263674

[bibr67-00131644261422169] Ziegler M. Maaß U. Griffith R. Gammon A. (2015). What is the nature of faking? Modeling distinct response patterns and quantitative differences in faking at the same time. Organizational Research Methods, 18(4), 679–703. 10.1177/1094428115574518

[bibr68-00131644261422169] Ziegler M. MacCann C. Roberts R. D. (2011). Faking: Knowns, unknowns, and points of contention. In Ziegler M. MacCann C. Roberts R. D. (Eds.), New perspectives on faking in personality assessment (pp. 3–16). Oxford University Press. 10.1093/acprof:oso/9780195387476.003.0011

