# Supplementary material for: Faking in High-Stakes Personality Assessments: A Response-Time-Based Latent Response Mixture Modeling Approach
Source: Educ Psychol Meas. 2026 Mar 18:00131644261422169. Online ahead of print. doi: 10.1177/00131644261422169 (PMC12999537; doi:10.1177/00131644261422169)
Supplement: sj-docx-1-epm-10.1177_00131644261422169 – Supplemental material for Faking in High-Stakes Personality Assessments: A Response-Time-Based Latent Response Mixture Modeling Approach [file sj-docx-1-epm-10.1177_00131644261422169.docx]

# Supplement

to the Manuscript

*Faking in High-Stakes Personality Assessments:*

*A Response-Time-Based Latent Response Mixture Modeling Approach*

## Details on the Data Generation in the Parameter Recovery Study

The purpose of the parameter recovery study of the present article was to examine a) how well the proposed model can recover model parameters under realistic conditions and b) to what extent less complex models are inferior regarding the recovery of model parameters. We therefore considered a data situation of a test measuring 3 substantive traits (10 items each, 7-point Likert scale), with a sample size of $N=500$.

To emulate a realistic ground truth for data generation, parameter estimates from the empirical demonstration of the proposed model were used as data-generating values. Figure S1 illustrates the distributions of data-generating values for the parameters of the item response, item response time (RT), and latent response model components. In addition, scoring weights of substantive trait dimensions were set to $\left( \begin{matrix} 0 & 1 & 2 & 3 & 4 & 5 & 6 \end{matrix} \right)$. Scoring weights of the faking dimension were set to the values from the empirical demonstration (collected by Seitz, Alagöz, & Meiser, 2025, Pilot Study 2; possible range from 0 to 6). Using the R package *MASS* (Venables & Ripley, 2002), true person parameter values were drawn from $MVN(\boldsymbol{\mu}, \boldsymbol{\Sigma})$, with $\boldsymbol{\mu}=\boldsymbol{0}$ and $\boldsymbol{\Sigma}$ as the matrix of estimated latent correlations from the empirical demonstration. The estimated class proportions from the empirical demonstration represented the true class proportions. For true class membership, values were sampled from $Cat(\boldsymbol{\pi}_{ni})$ using the *extraDistr* package (Wolodzko, 2023), with $\boldsymbol{\pi}_{ni}$ being a vector of person- and item-specific class probabilities computed from the latent response model (Equation 7 in the Main Text). Based on the true values of parameters from the different model components, true person parameters, as well as true class memberships, individual item responses and item response times (RT) were simulated using the class-specific categorical distributions of item responses (Equations 1 to 3) and log-normal distributions of item RTs (Equations 4 to 6). This procedure of data generation was replicated such that 50 independent datasets were simulated.

To each simulated dataset, the proposed person-by-item mixture model including RTs as well as four less complex models were fitted, namely a person-by-item mixture model not including RTs, a person mixture model including RTs, a person mixture model not including RTs, as well as a non-mixture model. Comparing models with and without RTs allowed investigating how accounting for RTs improves model estimation. Comparing person-by-item and person mixture models allowed investigating the consequences of assuming a constant strategy use per person. Comparing mixture and non-mixture models allowed examining the effect of assuming a single response strategy across persons and items. The results of the parameter recovery study are reported in the Main Text.

**Figure S1**

*Distributions of Data-Generating Values in the Parameter Recovery Study*

| a) Item slopes of substantive traits | b) Item slopes of faking | c) Item-category intercepts (“S-only class”) | d) Item-category intercepts (“S&F class”) | e) Item-category intercepts (“F-only class”) |
| --- | --- | --- | --- | --- |
| 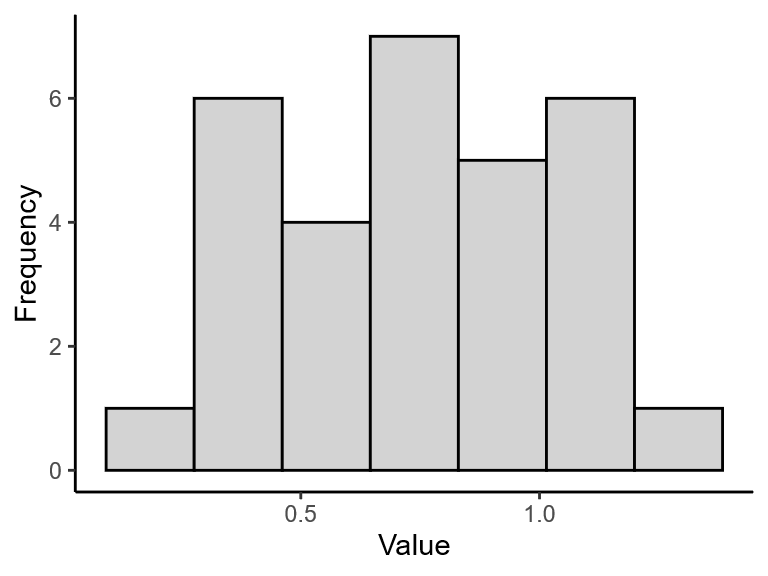 | 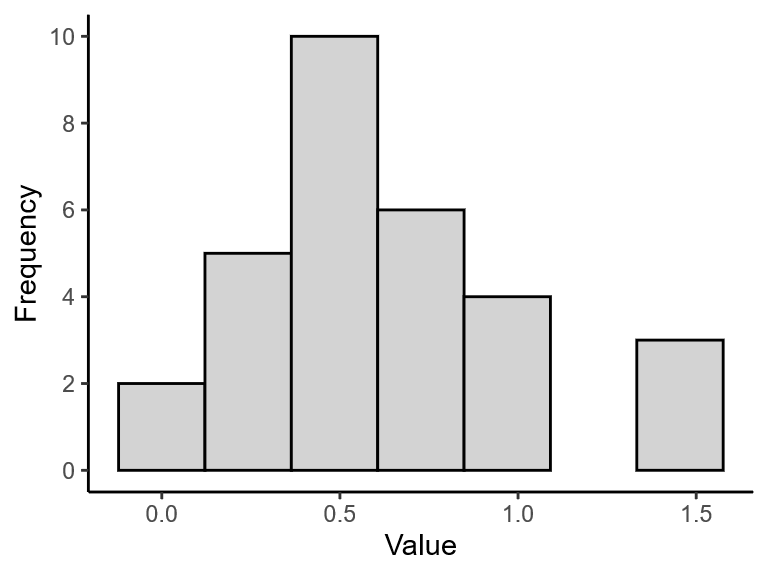 | 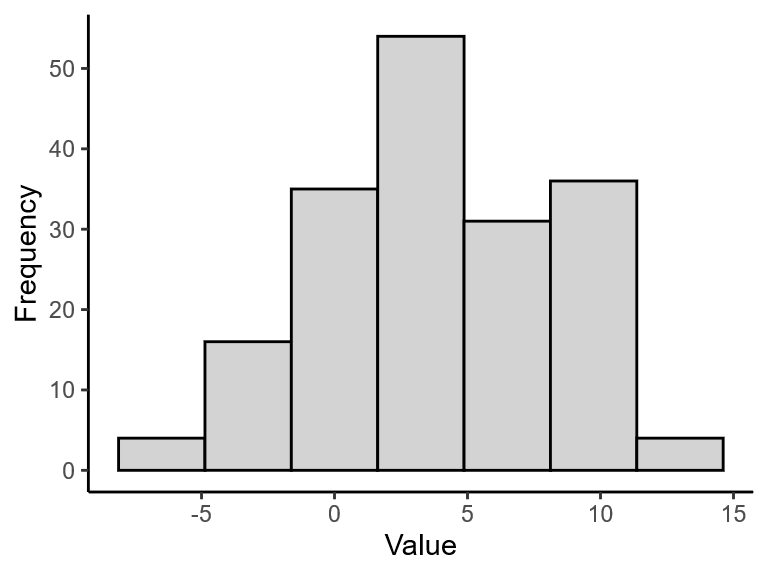 | 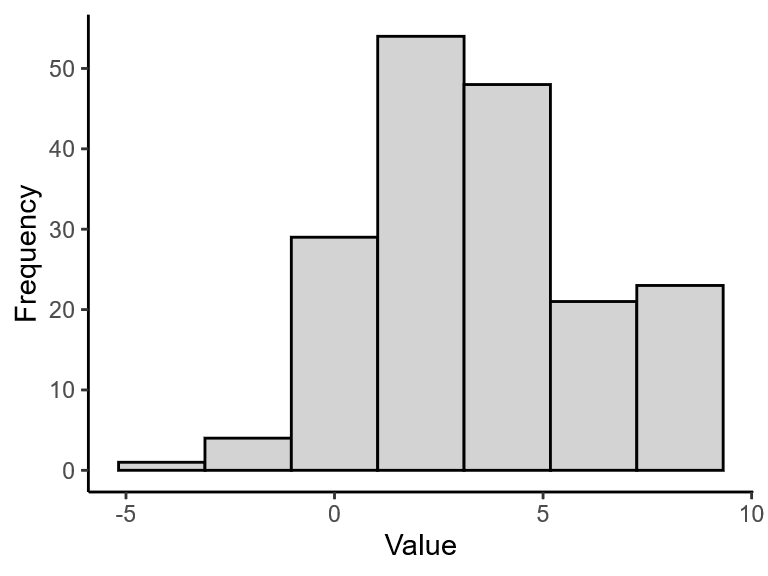 | 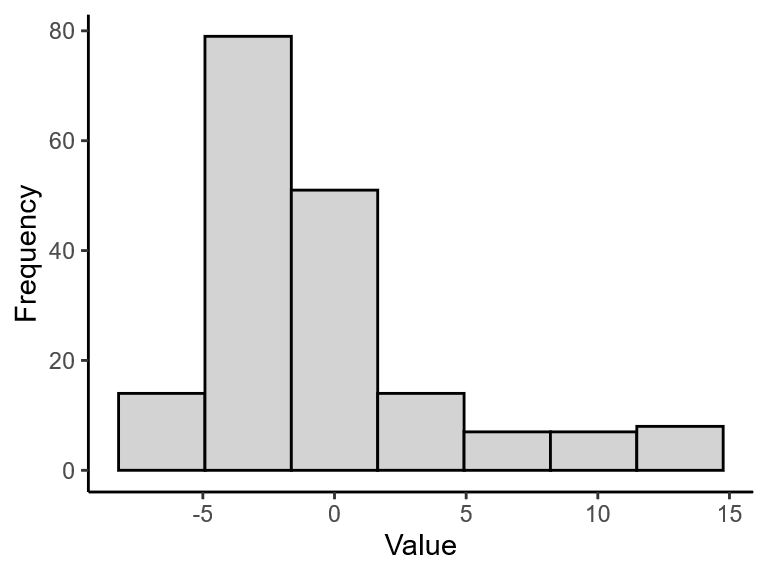 |
| f) Slope of speed | g) Item time intensities (“S-only class”) | h) Item time intensities (“F-only class”) | i) Proportionality constant | j) Residual SDs of log-RTs |
| $\text{ν}_{\text{φ}}\text{ = 0.21}$ | 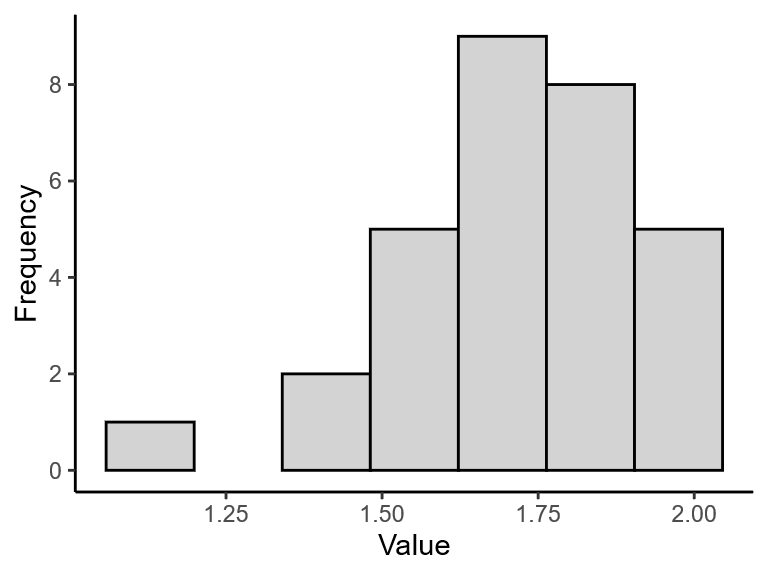 | 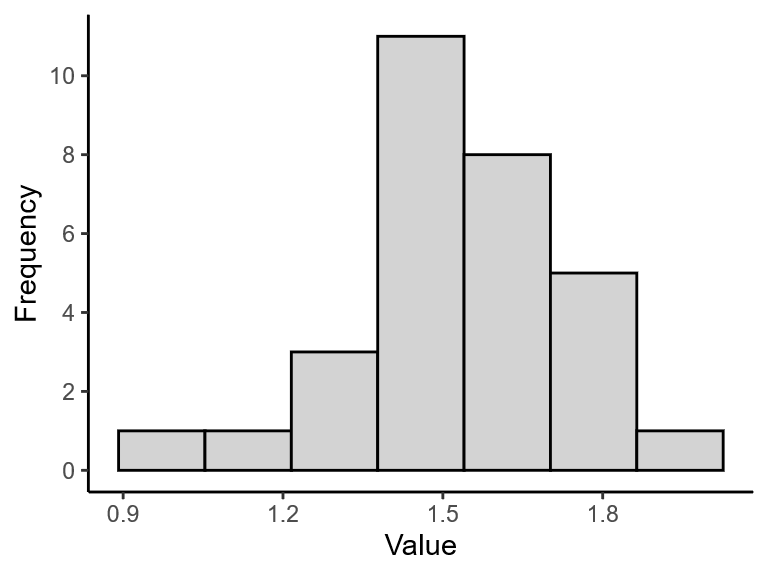 | $\text{λ = 0.23}$ | $\text{ς}_{\left( \text{S} \right)}\text{ = 0.23}$  $\text{ς}_{\left( \text{SF} \right)}\text{ = 0.43}$  $\text{ς}_{\left( \text{F} \right)}\text{ = 0.20}$ |
| k) Slope of strategy inclination | l) Item-class intercepts |  |  |  |
| $\text{ν}_{\text{ψ}}\text{ = 1.05}$ | 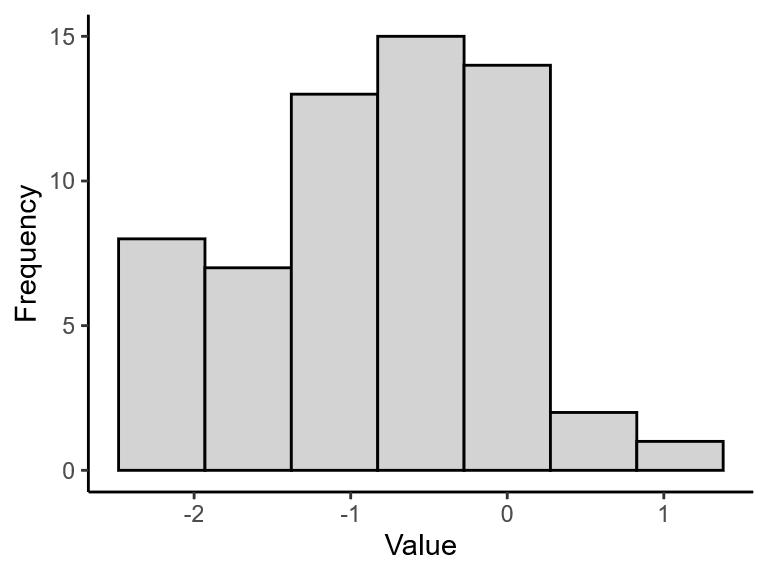 |  |  |  |

*Note.* Parameters in a to e are item response model parameters; parameters in f to j are item response time model parameters; parameters in k and l are latent response model parameters. Data-generating values for latent correlations are displayed in Table 2 in the Main Text. SD = standard deviation.

# References

Seitz, T., Alagöz, Ö. E. C., & Meiser, T. (2025). Disentangling qualitatively different faking strategies in high-stakes personality assessments: A mixture extension of the multidimensional nominal response model. *Educational and Psychological Measurement, 85*(6), 1237–1277. <https://doi.org/10.1177/00131644251341843>

Venables, W. N., & Ripley, B. D. (2002). *Modern applied statistics with S* (4^th^ ed.). Springer.

Wolodzko, T. (2023). *extraDistr: Additional univariate and multivariate distributions* (version 1.10.0) [Computer software]. <https://cran.r-project.org/web/packages/extraDistr/index.html>
